# What are the most efficacious treatment regimens for isoniazid-resistant tuberculosis? A systematic review and network meta-analysis

**DOI:** 10.1136/thoraxjnl-2015-208262

**Published:** 2016-06-13

**Authors:** H R Stagg, R J Harris, H-A Hatherell, D Obach, H Zhao, N Tsuchiya, K Kranzer, V Nikolayevskyy, J Kim, M C Lipman, I Abubakar

**Affiliations:** 1 Research Department of Infection and Population Health, University College London, London, UK; 2 Statistics, Modelling and Economics Department, Public Health England, London, UK; 3 UCL CoMPLEX, Faculty of Mathematics and Physical Sciences, University College London, London, UK; 4 Respiratory Diseases Department, National Infections Service, Public Health England, London, UK; 5 Department of Preventive Medicine and Epidemiology, Tohoku Medical Megabank Organization, Tohoku University, Sendai, Japan; 6 National and Supranational Mycobacterium Reference Laboratory, Research Centre Borstel, Borstel, Germany; 7 National Mycobacterium Reference Laboratory, Public Health England, London, UK; 8 Department of Medicine, Imperial College London, London, UK; 9 Division of Pulmonology and Critical Care Medicine, Department of Internal Medicine, College of Medicine, Incheon St. Mary's Hospital, The Catholic University of Korea, Seoul, South Korea; 10 UCL Respiratory, Division of Medicine, University College London, London, UK; 11 Royal Free London National Health Service Foundation Trust, London, UK; 12 MRC Clinical Trials Unit, University College London, London, UK

**Keywords:** Tuberculosis, Clinical Epidemiology

## Abstract

**Introduction:**

Consensus on the best treatment regimens for patients with isoniazid-resistant TB is limited; global treatment guidelines differ. We undertook a systematic review and meta-analysis using mixed-treatment comparisons methodology to provide an up-to-date summary of randomised controlled trials (RCTs) and relative regimen efficacy.

**Methods:**

Ovid MEDLINE, the Web of Science and EMBASE were mined using search terms for TB, drug therapy and RCTs. Extracted data were inputted into fixed-effects and random-effects models. ORs for all possible network comparisons and hierarchical rankings for different regimens were obtained.

**Results:**

12 604 records were retrieved and 118 remained postextraction, representing 59 studies—27 standalone and 32 with multiple papers. In comparison to a baseline category that included the WHO-recommended regimen for countries with high levels of isoniazid resistance (rifampicin-containing regimens using fewer than three effective drugs at 4 months, in which rifampicin was protected by another effective drug at 6 months, and rifampicin was taken for 6 months), extending the duration of rifampicin and increasing the number of effective drugs at 4 months lowered the odds of unfavourable outcomes (treatment failure or the lack of microbiological cure; relapse post-treatment; death due to TB) in a fixed-effects model (OR 0.31 (95% credible interval 0.12–0.81)). In a random-effects model all estimates crossed the null.

**Conclusions:**

Our systematic review and network meta-analysis highlight a regimen category that may be more efficacious than the WHO population level recommendation, and identify knowledge gaps where data are sparse.

**Systematic review registration number:**

PROSPERO CRD42014015025.

**Key messages**

**What is the key question?**

- What regimen is the most efficacious for treating patients with isoniazid-resistant TB?

**What is the bottom line?**

- In comparison to a baseline category that included the WHO's recommended regimen for countries with high levels of isoniazid resistance, this systematic review and meta-analysis identified that extending the duration of rifampicin and increasing the number of effective drugs present at 4 months increases efficacy.

**Why read on?**

- Treatment guidelines for isoniazid-resistant TB are diverse and lack consensus, despite the burden of such resistance globally; our systematic review summarises the evidence behind recommending different regimens.

## Introduction

In 2014, 3.3% of new patients with TB globally and 20% of those previously treated had multidrug-resistant (MDR) TB, that is, resistance to both of the first-line drugs isoniazid (INH) and rifampicin (RIF).^1^ Outside of Eastern Europe 13.9% of incident disease was estimated to be INH-resistant 1994–2009, but 44.9% within Eastern Europe.^2^ The loss of INH, a drug with a relatively low risk of adverse events (AEs) and potent early bactericidal activity, would compromise the treatment of active TB.^2^ On an individual level, patients with INH-monoresistant disease are at a theoretically greater risk of developing MDR than those with drug-sensitive TB due to the requirement for only a single additional resistance mutation, with the associated risk of a need for more expensive, toxic and lengthy treatment regimens.^3^ At a population level, inadequate treatment of monoresistant disease leading to an increased prevalence of MDR could be highly detrimental for TB control programmes.

In countries with ‘high’ levels of INH resistance in new patients with TB, the WHO has recommended a 2 month intensive phase of INH, RIF, pyrazinamide (PZA) and ethambutol (EMB) followed by a 4 month continuation phase of INH, RIF and EMB in patients without INH susceptibility testing or where results are not available before the continuation phase.^3^ If detailed individual-level drug susceptibility results are accessible more comprehensive recommendations are made, for example, 6–9 months of RIF, PZA and EMB (plus or minus a fluoroquinolone) for INH-monoresistant or INH and streptomycin (STM)-resistant disease.^4^ The American Thoracic Society (ATS) recommends that INH-resistant TB is treated with a 6 month regimen of RIF, PZA and EMB (plus a fluoroquinolone for extensive disease).^5^ The National Institute for Health and Care Excellence (NICE), UK, recommends a 9 month regimen (10 months where disease is extensive) of 2 months of RIF, PZA and EMB then 7 months of RIF and EMB.^6^ All three bodies recognise the need for further research in this area.

Conventional meta-analyses only allow direct comparisons between regimens contrasted within specific studies and are highly limited in the inferences they can make about relative efficacy. Previous reviews of INH-resistant TB treatment have been restricted by the methodology available.^7–9^ By comparison, Bayesian hierarchical models use a network approach that generates indirect comparisons of regimens for incorporation into inferences of relative efficacy.^10^ ^11^ We undertook a systematic review and meta-analysis using this approach to provide a vital updated evidence summary of randomised controlled trials (RCTs) of the treatment of non-MDR INH-resistant TB (referred to throughout as ‘INH resistant’),^6^ and to assess relative regimen efficacy at preventing negative outcomes (treatment failure or the lack of microbiological cure; relapse post-treatment; death due to TB).

## Methods

### Data sources and searches

Ovid MEDLINE, the Web of Science and EMBASE were mined using search terms for TB, drug therapy and RCTs (see online supplementary file 1). Reference lists of included papers and review articles were also searched. This review was registered with PROSPERO-CRD42014015025.

10.1136/thoraxjnl-2015-208262.supp1 Supplementary file 1

### Study selection

*Inclusion criteria*:

- RCTs indexed by the 21st of January 2015 of antimicrobial regimens for TB disease
- RCT used culture to confirm disease and drug sensitivity tested
- Treatment outcomes and/or relapses post-treatment extractable either specifically for patients with INH-resistant strains or for the entire study population if ≥85% of that population had INH-resistant disease
- All INH resistance profiles retained, provided strains not MDR
- No language restrictions

*Exclusion criteria*:

- Trials in animals
- Trials that were not RCTs comparing at least two antimicrobial regimens

HRS screened all (and H-AH 10%) of the retrieved records from the de-duplicated titles to full texts. H-AH also independently undertook the final stage of full text screening of all articles identified as potentially being includable by HRS. Where consensus was not achieved a third reviewer (IA) was available to resolve discrepancies.

### Data extraction and quality assessment

Two reviewers (HRS and H-AH) independently extracted publications into a standardised predesigned spreadsheet (see online supplementary file 2). Discrepancies were resolved by discussion and study authors contacted if necessary. Publications not written in a language fluently spoken by HRS or H-AH were extracted by an additional reviewer.

10.1136/thoraxjnl-2015-208262.supp2 Supplementary file 2

HRS and H-AH independently assessed study quality using the Cochrane Collaboration's tool for evaluating bias (see online supplementary file 2).^12^ A sensitivity analysis excluding studies deemed at high risk of bias across all domains was planned.

### Data synthesis

**Figure 1:**
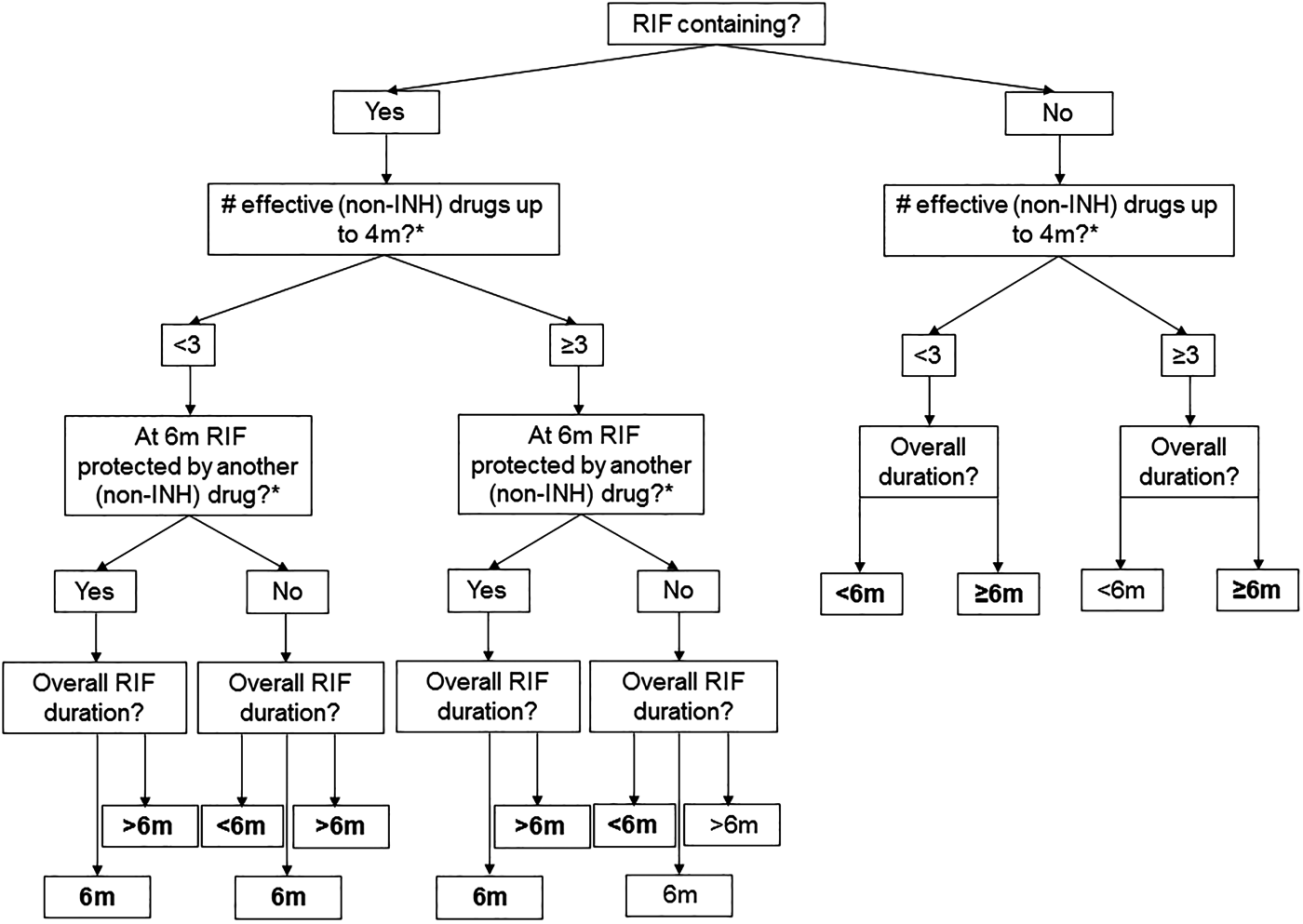
Treatment regimen categorisation. Regimen categorisation flow chart for main analysis. Second question includes RIF in the calculation. Bolded text after final question indicates regimen category (table 1) present in main network (figure 3B). *Levamisole and diphenyl thiourea compound SU 1906 not counted as effective protection. INH, isoniazid; RIF, rifampicin.

Treatment regimens were categorised via a decision tree that reflected the roles of particular drugs, the importance of RIF and treatment duration (figure 1 and table 1). The protection of RIF by another effective drug (ED) was assessed at 6 months to reflect the usual treatment length for drug-sensitive cases. In the absence of a consistent definable intensive phase across regimens we chose to assess the number of EDs at 4 months, double the usual intensive phase length in drug-sensitive treatment regimens. If regimens were shorter than 6 months the drugs at the end were considered for the question ‘was RIF protected by another drug at six months?’

**Table 1:** Regimen category codes

RIF containing?	# of effective drugs (ED) up to 4m?	At 6m RIF protected (Pr6) by another effective drug?	Overall (RIF) duration (D)	Code
No	<3	N/A	<6m	ED<3 D<6m
No	<3	N/A	≥6m	ED<3 D≥6m
No	≥3	N/A	≥6m	ED≥3 D≥6m
Yes	<3	No	<6m	RIF ED<3 D<6m
Yes	<3	No	6m	RIF ED<3 D=6m
Yes	<3	No	>6m	RIF ED<3 D>6m
Yes	<3	Yes	6m	RIF ED<3 Pr6 D=6m
Yes	<3	Yes	>6m	RIF ED<3 Pr6 D>6m
Yes	≥3	No	<6m	RIF ED≥3 D<6m
Yes	≥3	Yes	6m	RIF ED≥3 Pr6 D=6m
Yes	≥3	Yes	>6m	RIF ED≥3 Pr6 D>6m

Encoded regimen categories (figure 1) present in the main network.

4m, 4 months; 6m, 6 months; D</=/>6m, overall RIF duration or duration of the entire regimen if RIF not present; N/A, not applicable; Pr6, protected at 6 months; RIF, rifampicin.

A composite negative outcome of (1) death due to TB, (2) treatment failure (a lack of clinical improvement necessitating a regimen change) or no microbiological cure and (3) relapse post-treatment was generated. Cures and relapses were preferably defined by culture conversion. AEs were extracted (see online supplementary file 2), but not reported consistently enough to be included.

### Statistical analysis

Direct evidence was initially analysed using a standard pairwise meta-analysis in Stata V.13.1 using metan (see online supplementary file 3).^13^ Similar to that mentioned earlier,^14^ a mixed-treatment comparisons (MTC) approach was then used, which extends standard meta-analysis to multiple treatments. Briefly, fixed-effects and random-effects models as described by Dias *et al*^15^ were fitted within a Bayesian framework, producing point estimates, 95% credible intervals (CrI) and treatment rankings (see online supplementary file 3, which also documents our approach to assess network inconsistency and publication bias).

10.1136/thoraxjnl-2015-208262.supp3 Supplementary file 3

## Results

Post-deduplication 12 604 records were retrieved (see online supplementary file 4). A 98% consensus was achieved during double screening. After full text extraction (see online supplementary file 5) 118 were included, representing 59 studies—27 standalone and 32 with multiple papers.^16–74^ In the latter instance baseline data were usually taken from the earliest publication and relapses from the latest.

10.1136/thoraxjnl-2015-208262.supp4 Supplementary file 4

10.1136/thoraxjnl-2015-208262.supp5 Supplementary file 5

Forty-three studies provided data on patients with TB with non-MDR INH-monoresistant strains,^17–19^ ^21–36^ ^38–42^ ^44–48^ ^50–52^ ^56^ ^57^ ^60–62^ ^64^ ^66^ ^68^ ^71^ ^73^ ^74^ 36 on patients with TB with INH-resistant and STM-resistant strains^16^ ^19–25^ ^27–38^ ^40^ ^42–45^ ^47^ ^49^ ^51–53^ ^59^ ^60^ ^62^ ^63^ ^65^ ^66^ and eight on more complex non-MDR resistance patterns (see online supplementary file 6).^26^ ^54^ ^55^ ^58^ ^67^ ^69^ ^70^ ^72^ One study contained INH and p-aminosalicylic acid-resistant patients that were not extracted as one treatment arm was composed solely of these two drugs.^64^ In five studies the results extracted were not for pure INH-resistant populations, but the proportion resistant to INH was above our threshold of 85% and not substantially different by arm.^43^ ^50^ ^69^ ^72^ ^74^

10.1136/thoraxjnl-2015-208262.supp6 Supplementary file 6

Forty-five studies were not solely focused on patients with drug-resistant TB.^17–26^ ^28–42^ ^44^ ^45^ ^47^ ^51–54^ ^56–64^ ^66^ ^67^ ^71^ ^73^ One had specific inclusion criteria for extrapulmonary (abdominal) disease.^17^ No studies were conducted solely in children; one looked specifically at HIV-positive individuals.^58^ One did not randomise at the individual level.^51^ Ten contained more than 100 analysable patients with INH-resistant disease^16^ ^18^ ^20^ ^27^ ^43^ ^46^ ^47^ ^49^ ^69^ ^70^ and 29 over 50.^16^ ^18^ ^20^ ^27^ ^29^ ^30^ ^35^ ^36^ ^38^ ^40^ ^42–49^ ^51^ ^55^ ^57^ ^60^ ^65^ ^66^ ^68–72^ Two reported data before patients had completed the full length of assigned treatment; we recorded regimen length appropriately.^60^ ^71^ Twenty-two did not assess relapse post-treatment.^17^ ^21^ ^37^ ^42^ ^46^ ^47^ ^49^ ^50^ ^53–55^ ^59^ ^60^ ^63–65^ ^67^ ^68^ ^70^ ^71^ ^73^ ^74^ Follow-up time after the end of treatment ranged from 3 months to 7.5 years. The majority of studies reporting relapses defined them on the basis of culture status (usually two or more positive cultures in a 3-month period).

### Quality

Across four of the six quality domains (randomisation, allocation concealment, blinding of participants and personnel, blinding of outcome assessment) many of RCTs did not record sufficient information to assess the risk of bias (see online supplementary file 7). One study was considered at high risk due to lacking allocation concealment,^27^ five lacked blinding of participants and personnel (four of these did not blind outcome assessment)^17^ ^19^ ^57^ ^58^ ^69^ and 40 had high levels of attrition (linked to the length of follow-up).^18^ ^22–29^ ^31^ ^33^ ^34^ ^36–40^ ^42–47^ ^50^ ^51^ ^53^ ^55–57^ ^60–62^ ^64^ ^66–70^ ^72^ ^74^ No studies were at high risk of bias across all domains.

10.1136/thoraxjnl-2015-208262.supp7 Supplementary file 7

### Treatment of patients with INH-resistant TB

**Figure 2:**
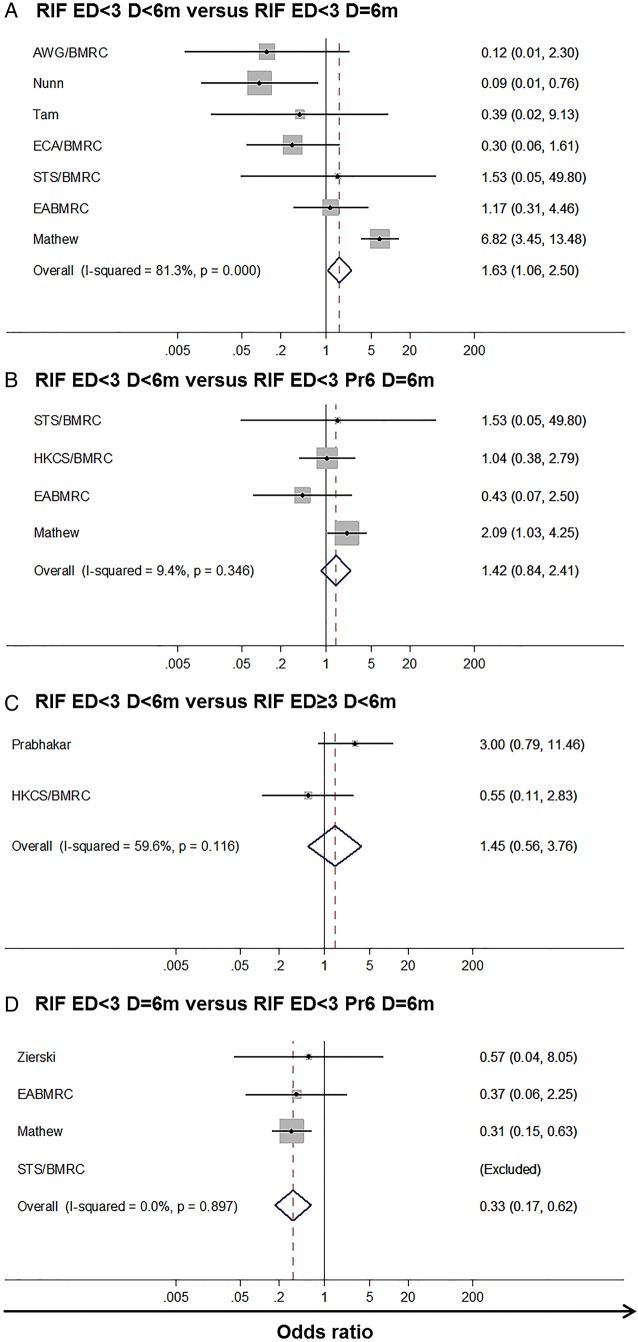
Pairwise direct effects forest plots across all isoniazid resistance profiles. Pairwise direct effects forest plots for the four regimen pairs where such comparisons were possible. Regimen RIF ED<3 D<6m the baseline for plots (A–C) and regimen RIF ED<3 D=6m for plot (D). Regimen (A) RIF ED<3 D=6m, (B) RIF ED<3 Pr6 D=6m, (C) RIF ED≥3 D<6m, (D) RIF ED<3 Pr6 D=6m the comparator. In analysis (D) study STS/BMRC had no events in either arm. Vertical solid line—null hypothesis. Vertical dotted line summary estimate. AWG/BMRC, Algerian Working Group/British Medical Research Council Cooperative Study; EABMRC, East African British Medical Research Council Study; ECA/BMRC, East and Central African/British Medical Research Council; ED, effective drugs; HKCS/BMRC, Hong Kong Chest Service/British Medical Research Council; RIF, rifampicin; STS/BMRC, Singapore TB Service/British Medical Research Council.

Data were available for 11 of a potential 14 regimen categories (figure 1 and table 1). Regimens were categorised as per table 1, for example, RIF ED<3 Pr6 D>6m represents a RIF-containing regimen using fewer than three ED at 4 months, where RIF was protected by an ED at 6 months (Pr6), and the overall duration (D) of RIF was greater than 6 months. Using data from any INH resistance pattern, few pairwise inferences were available of the relative efficacy (figure 2). In two comparisons one study showed outlying results.^20^ Considerable heterogeneity was observed for one comparison, likely due to the same study, otherwise fixed effects were deemed appropriate.

**Figure 3:**
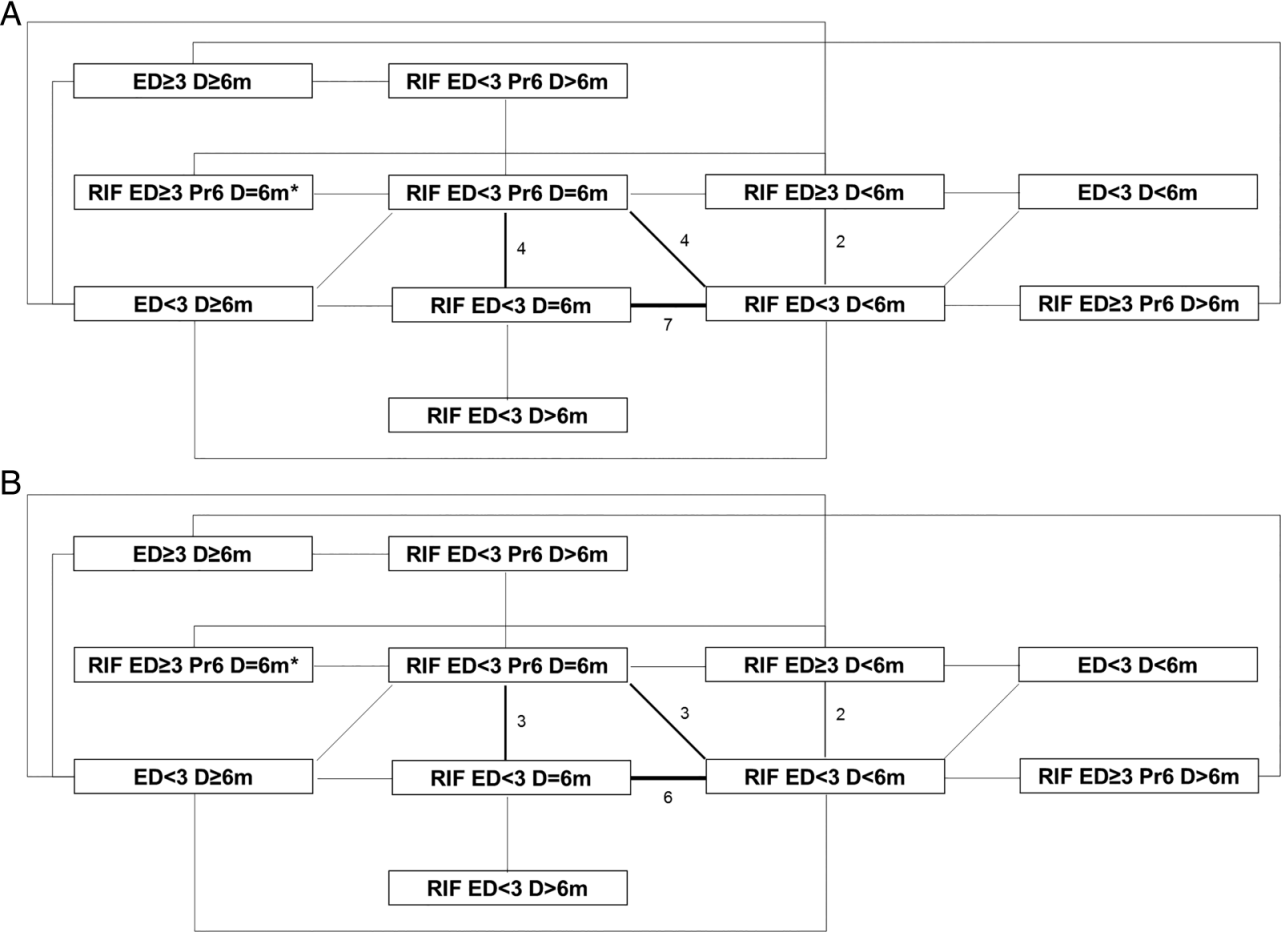
Data network. Data networks (A) for the main analysis containing all isoniazid resistance patterns, (B) for the main analysis excluding the inconsistent study. Thickness of lines and numbers indicate the number of studies making this comparison. *One study arm classifiable as RIF ED≥3 Pr6 D=6m, RIF ED<3 Pr6 D=6m or RIF ED<3 D=6m compared with RIF ED<3 D=6m; here listed as RIF ED≥3 Pr6 D=6m as per main analysis. ED, effective drugs; RIF, rifampicin.

**Figure 4:**
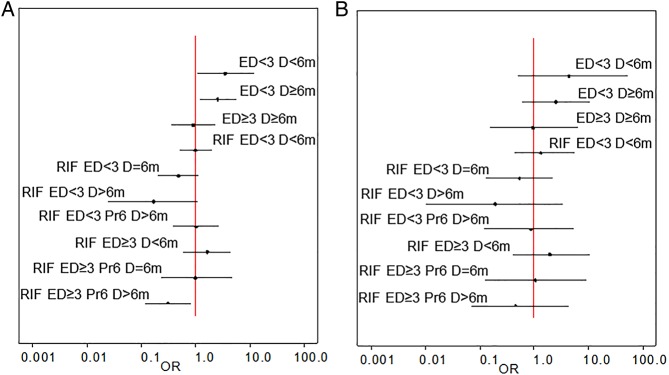
Forest plots from fixed-effects and random-effects network meta-analyses across all isoniazid resistance profiles. Forest plots of treatment comparisons from the network depicted in figure 3B. (A) Fixed-effects and (B) random-effects derived ORs on a log scale with 95% credible interval. Vertical line—null hypothesis. ED, effective drugs; RIF, rifampicin.

**Figure 5:**
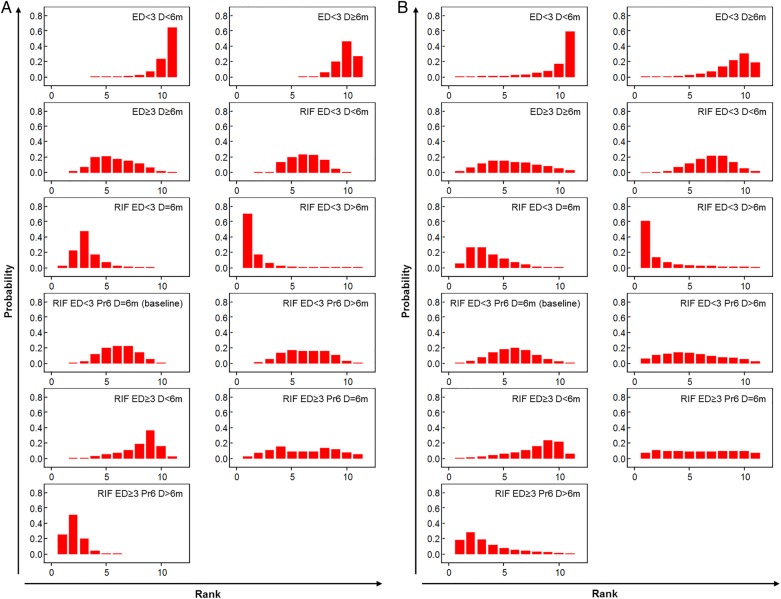
Histograms of relative ranks from fixed-effects and random-effects network meta-analyses across all isoniazid resistance profiles. Relative treatment ranks of treatment comparisons from the network depicted in figure 3B. (A) Fixed effects. (B) Random effects. ED, effective drugs; RIF, rifampicin.

Given such limitations we undertook a network meta-analysis. Regimen category RIF ED<3 Pr6 D=6m, in which the WHO-recommended regimen for countries with high levels of INH resistance sits, was set as the network baseline (figure 3A); 12 studies were included in this category. Model fitting proceeded as described in online supplementary file 8; one inconsistent study,^20^ identified as an outlier in the pairwise analysis, was excluded (figure 3B). Results are presented from both fixed-effects and random-effects models (table 2 and figures 4 and 5). In the fixed-effects model regimen category RIF ED≥3 Pr6 D>6m (present in two studies) was predicted to have a lower likelihood of a negative outcome than category RIF ED<3 Pr6 D=6m (OR 0.31 (95% CrI 0.12–0.81)); RIF ED<3 D>6m and RIF ED<3 D=6m also had low OR estimates, but their CrIs crossed the null (0.17 (0.02–1.07) and 0.48 (0.20–1.14), respectively) (table 2). In the random-effects model all effect estimates crossed the null. Categories RIF ED<3 D>6m, RIF ED<3 D=6m and RIF ED≥3 Pr6 D>6m ranked highest in the fixed-effects model, although with substantial uncertainty for the former (figure 5A). This held true in the random-effects model (figure 5B). Absolute differences in the proportion of patients with a negative outcome (table 2) showed that in the fixed-effects model RIF ED≥3 Pr6 D>6m reduced the baseline proportion of 0.19 by 0.12, that is, approximately seven remaining negative outcomes per 100 (equivalent change in random-effects model 16 patients to 8).

**Table 2:** ORs, relative ranks and absolute proportion difference from fixed-effects and random-effects network meta-analyses across all isoniazid resistance profiles

	OR (95% CrI)		Rank (95% CrI)		Proportion difference (95% CrI)	
Treatment	Fixed effects	Random effects	Fixed effects	Random effects	Fixed effects	Random effects
ED<3 D<6m	3.47 (1.08–11.55)	4.38 (0.50–52.41)	11 (8–11)	11 (4–11)	0.26 (0.01–0.52)	0.29 (−0.09–0.74)
ED<3 D≥6m	2.54 (1.19–5.59)	2.50 (0.62–10.36)	10 (8–11)	10 (5–11)	0.18 (0.03–0.34)	0.16 (−0.07–0.43)
ED≥3 D≥6m	0.91 (0.35–2.30)	0.95 (0.15–6.21)	6 (3–9)	6 (2–10)	−0.01 (−0.14–0.14)	−0.01 (−0.20–0.31)
RIF ED<3 D<6m	1.00 (0.52–1.95)	1.32 (0.44–5.52)	6 (4–9)	7 (4–10)	0.00 (−0.11–0.10)	0.04 (−0.13–0.25)
RIF ED<3 D=6m	0.48 (0.20–1.14)	0.53 (0.13–2.20)	3 (2–6)	3 (1–7)	−0.09 (−0.20–0.02)	−0.07 (−0.23–0.09)
RIF ED<3 D>6m	0.17 (0.02–1.07)	0.19 (0.01–3.36)	1 (1–6)	1 (1–9)	−0.15 (−0.26–0.01)	−0.12 (−0.28–0.19)
RIF ED<3 Pr6 D=6m	Baseline	Baseline	6 (3–9)	6 (2–9)	Baseline	Baseline
RIF ED<3 Pr6 D>6m	1.01 (0.39–2.62)	0.85 (0.12–5.25)	6 (3–10)	5 (1–11)	0.00 (−0.13–0.17)	−0.02 (−0.20–0.30)
RIF ED≥3 D<6m	1.60 (0.59–4.35)	1.89 (0.40–10.27)	9 (4–10)	9 (3–11)	0.08 (−0.08–0.28)	0.10 (−0.13–0.43)
RIF ED≥3 Pr6 D=6m	0.98 (0.24–4.57)	1.04 (0.13–9.25)	6 (2–11)	6 (1–11)	0.00 (−–0.16–0.31)	0.01 (−0.19–0.44)
RIF ED≥3 Pr6 D>6m	0.31 (0.12–0.81)	0.45 (0.07–4.32)	2 (1–4)	3 (1–9)	−0.12 (−0.23–0.02)	−0.08 (−0.24–0.23)

Main results from the network depicted in figure 3B. Baseline proportion with a negative outcome 0.19 (95% CrI 0.12–0.29) in the fixed-effects model and 0.16 (95% CrI 0.07–0.31) in the random-effects model.

CrI, credible interval; ED, effective drugs; Pr6, protected at 6 months; RIF, rifampicin.

Sensitivity analyses were undertaken to examine the impact of changing the regimen categorisation of a single arm of one study where grouping was uncertain as reported outcomes were not fully separated by PZA usage (see online supplementary file 10).^66^ Relatively little impact on the effect estimates was observed.

In a network adjusted for the dose of RIF, RIF ED≥3 Pr6 D>6m remained the only category in the fixed-effects model where the odds of a negative outcome were lower than that of the baseline and did not cross the null (0.22 (0.06 to 0.75)) (see online supplementary file 11). The impact of taking a lower dose of RIF was not consistent, with a high degree of uncertainty.

The relative efficacy of different regimens may change if there is additional drug resistance present. Restricting our analysis to patients with INH-monoresistant strains left 43 studies, of which 23 tested the same regimen group in all arms and a further three had no events (see online supplementary file 12). Of the remaining 17 studies 11 had arms condensed together. In both models uncertainty was very great due to data sparsity (eg, one study examined RIF ED≥3 Pr6 D>6m), preventing firm conclusions.

10.1136/thoraxjnl-2015-208262.supp8 Supplementary file 8

10.1136/thoraxjnl-2015-208262.supp9 Supplementary file 9

10.1136/thoraxjnl-2015-208262.supp10 Supplementary file 10

10.1136/thoraxjnl-2015-208262.supp11 Supplementary file 11

10.1136/thoraxjnl-2015-208262.supp12 Supplementary file 12

### Inconsistency

Effect estimates from both fixed-effects and random-effects pairwise meta-analyses without the inconsistent study were compared with those from the random-effects MTC to evaluate if evidence was systematically inconsistent or simply randomly variable. Only two pairwise comparisons were made by more than two studies. Estimates from both were highly similar to those from the random-effects network meta-analysis, suggesting limited detectable inconsistency: RIF ED<3 D<6m versus RIF ED<3 D=6m—fixed pairwise 0.36 (95% CI 0.17 to 0.77), random pairwise 0.41 (95% CI 0.16 to 1.05), random network 0.41 (95% CrI 0.11–1.10); RIF ED<3 D<6m versus RIF ED<3 Pr6 D=6m—fixed pairwise 0.85 (95% CI 0.37 to 1.95), random pairwise 0.87 (95% CI 0.38 to 2.01), random network 0.76 (95% CrI 0.18–2.28).

### Publication bias

Little evidence was seen of small-study effects for the four possible pairwise comparisons with the inconsistent study included (see online supplementary file 13). The Harbord test was undertaken where a comparison was made by more than two studies: p value 0.07 for RIF ED<3 D<6m versus RIF ED<3 D=6m, 0.38 for RIF ED<3 D<6m versus RIF ED<3 Pr6 D=6m.

10.1136/thoraxjnl-2015-208262.supp13 Supplementary file 13

## Discussion

This is the first systematic review for non-MDR INH-resistant TB to use a MTC methodology to infer the relative efficacy of different treatment regimens. When studies of patients with strains of any non-MDR INH resistance profile were included in our network, regimen category RIF ED≥3 Pr6 D>6m (a RIF-containing regimen using three or more ED at 4 months, in which RIF is protected at 6 months, and RIF was taken for more than 6 months) appeared better than RIF ED<3 Pr6 D=6m (a RIF-containing regimen using fewer than three ED at 4 months, in which RIF was protected at 6 months, and RIF was taken for 6 months; category includes WHO-recommended regimen for countries with a high burden of INH resistance). In a network restricted to patients with INH-monoresistant disease data sparsity made conclusions difficult to draw.

Our network meta-analysis was constrained by the number of RCTs documenting outcomes for patients with INH-resistant disease and the number of individuals within these RCTs. Additionally, Bayesian methodologies have their own limitations when using sparse data. Indeed, the regimen rankings and effect estimates observed from our MTC are not fully consistent with what could be predicted from our regimen categories; data availability will play a major role here. Such a categorisation process was necessary, however, to allow the MTC approach. The uncertainty of our estimates is acknowledged, particularly with the random-effects models. We could not adjust for clustering in the cluster randomised study due to the nature of the data presented in that publication,^51^ although we expect the impact of this to be relatively small. We did not specifically stratify for the dose of particular drugs aside from RIF during our analysis; however, dosing was generally consistent across included RCTs.

The nature of the included studies introduced specific limitations. When INH monoresistance was reported resistance testing was not necessarily performed for other drugs. Relative levels of INH resistance were only reported in one study.^73^ INH resistance mutations were not reported, which was unfortunate given observational reports associating, for example, *katG_315_* with negative treatment outcomes.^75^ Extraction of intention-to-treat data was not possible for all studies. Some reported results specifically for unfavourable outcomes as defined, and in others this was calculated using data for favourable outcomes, which were not necessarily the opposite. Not all studies reported all three of the negative outcomes assessed; outcomes were not always culture confirmed. Data on AEs were minimal. We could not stratify by treatment adherence with the data available. The absence of significant numbers of studies in HIV-positive patients, children and individuals with extrapulmonary disease meant that restricted analysis examining these particular patient populations were not possible; this could introduce bias to the network if particular drug regimens were only trialled in specific settings.

In 1986, Mitchison and Nunn^9^ undertook a review of 12 (11 published) British Medical Research Council RCTs, which had tested different treatment regimens in patients with pulmonary TB with initial drug resistance. In the absence of a formal meta-analysis, they concluded that the sterilising ability of a regimen was not substantially altered in individuals with initial resistance to INH or STM. The number of drugs in the regimen and duration of RIF were thought to be influential, for example four to five drugs including (INH and) RIF in a 6 month regimen was deemed beneficial. Our review included all of these published studies and our results were not dissimilar.

Of the two Menzies *et al*^8^ systematic reviews published in 2009 the first, which sought to determine the effectiveness of the WHO-recommended retreatment regimen at that time using meta-regression, is more similar to our analysis. This study found no RCTs of the retreatment regimen, but concluded that, when considering the incidence rate of treatment failure or relapse, a RIF duration of 2 months or less, having few drugs in the intensive phase, and having therapy delivered twice weekly throughout worsened both outcomes. In comparison, our systematic review encompassed a highly dissimilar set of publications (including new RCTs and an update) and our differing analytical technique may have reduced the likelihood of bias (see online supplementary file 14). Even so, our results were similar in terms of the impact of RIF duration and the number of drugs present early in a regimen.

10.1136/thoraxjnl-2015-208262.supp14 Supplementary file 14

As a baseline for our meta-analysis we chose the category that contained the recommended WHO regimen for countries with high levels of INH resistance in new patients with TB to ascertain if there are more efficacious regimens. Within this category our review contained one study that evaluated this regimen (although with slightly altered daily doses and in abdominal TB), in which no INH-resistant patients had a negative outcome.^17^ The NICE-recommended regimen would be grouped as RIF ED<3 Pr6 D>6m within our groupings and the ATS regimen as RIF ED≥3 Pr6 D=6m; these two categories obtained middling ranks in our analysis, but we did not find includable studies specifically of either. We did not choose the WHO-recommended regimen for INH-monoresistant or INH-resistant and STM-resistant disease where individual-level drug susceptibility patterns are known as our baseline due to the lack of such testing in many countries. Although no studies specifically tested this regimen it sits either in category RIF ED≥3 Pr6 D=6m or RIF ED≥3 Pr6 D>6m, the latter of which was the most efficacious in our network. RIF ED≥3 Pr6 D>6m contained two trialled regimens: (1) seven and a half months of INH, STM, RIF and PZA administered daily for the first month and a half and then intermittently (apart from the INH) and (2) 12 months of EMB, morphazinamide (a drug closely related to PZA with more unfavourable AE profile)^76^ ^77^ and RIF, administered daily.

Ultimately, decisions as to the best regimen to use within a particular country will also depend on drug availability, cost and AEs. Category RIF ED≥3 Pr6 D>6m encompasses a variety of regimens; those reported here contain relatively cheap drugs, but also lengthy periods of components associated with a high likelihood of AEs. Precise recommendations can thus not be made without additional studies within this category. Regimens that do not use any INH, for example those trialled in the recent Rifaquin study, could also be useful for effective treatment.^78^

As Xpert MTB/RIF is rolled out globally many countries may switch to only undertaking further drug sensitivity testing on strains found to be genotypically RIF resistant. As such, non-MDR INH resistance may be increasingly underdiagnosed, and thus generally treated with the short-course regimen 2 months of INH EMB PZA RIF followed by 4 months of INH RIF. In our network this regimen falls into the category RIF ED<3 D=6m, for which the CrI in all models overlapped the null.

In our systematic review and network meta-analysis, against a baseline category of RIF-containing regimens with less than three ED at 4 months, where RIF was protected at 6 months and RIF was taken for 6 months, we demonstrate the efficaciousness of extending the duration of RIF and increasing the number of ED at 4 months, with a potential reduction in negative outcomes of ∼70%. By undertaking this work, we have identified further efficacious regimens for INH-resistant TB, a target of future research listed by WHO in their treatment guidance.^3^ Although more evidence was found for efficacious regimens than during our companion systematic review of treatment regimens for RIF-monoresistant disease,^79^ we clearly demonstrate the need for further studies of non-MDR INH-resistant TB specifically in HIV-positive individuals and children, as well as the efficacy of EMB in such regimens.^3^ Indeed, we agree with NICE that ‘[r]andomised controlled trials are needed to compare different anti-TB regimens for isoniazid-resistant TB, assessing mortality, treatment success or treatment failure, rates of relapse and adverse events’,^6^ and the ATS that ‘[d]efinitive randomized or controlled studies have not been performed among patients with… various patterns of drug resistance’.^5^
