# Supplementary material for: What are the most efficacious treatment regimens for isoniazid-resistant tuberculosis? A systematic review and network meta-analysis
Source: Thorax. 2016 Jun 13;71(10):940–9. doi: 10.1136/thoraxjnl-2015-208262 (PMC5036252; doi:10.1136/thoraxjnl-2015-208262)
Supplement: Supplementary file 1 [file thoraxjnl-2015-208262supp_file1.pdf]

**SUPPLEMENTARY FILE 1: Search strategy**

**Ovid MEDLINE(R) In-Process & Other Non-Indexed Citations** and **Ovid MEDLINE(R)**

| | | |
|---|---|---|
| #1 | exp Tuberculosis/ | 159,835 |
| #2 | (TB or tuberculo*).mp. [mp=title, abstract, original title, name of substance word, subject heading word, keyword heading word, protocol supplementary concept word, rare disease supplementary concept word, unique identifier] | 218,140 |
| #3 | 1 OR 2 | 219,653 |
| #4 | exp Drug Therapy/ | 1,059,465 |
| #5 | (treatment or therapy or therapeutic*).mp. [mp=title, abstract, original title, name of substance word, subject heading word, keyword heading word, protocol supplementary concept word, rare disease supplementary concept word, unique identifier] | 4,638,973 |
| #6 | 4 OR 5 | 5,056,475 |
| #7 | exp controlled clinical trial/ | 88,431 |
| #8 | exp Randomized Controlled Trial/ | 381,471 |
| #9 | randomi#ed.mp. | 600,633 |
| #10 | trial.mp. | 875,289 |
| #11 | 7 OR 8 OR 9 OR 10 | 1,026,177 |
| **#12** | **3 AND 6 AND 11** | **3,176** |

**Web of Science**

| | | |
|---|---|---|
| #1 | (Tuberculosis[MeSH Terms]) OR TB OR tuberculo* | ~619,973 |
| #2 | (Drug Therapy[MeSH Terms]) OR treatment OR therapy OR therapeutic* | ~21,168,564 |
| #3 | (Controlled Clinical Trial[MeSH Terms]) OR (Randomized Controlled Trial[MeSH Terms]) OR randomized OR randomised OR trial | ~3,704,538 |
| **#4** | **1 AND 2 AND 3** | **6,828** |

**Embase Classic+Embase**

| | | |
|---|---|---|
| #1 | exp tuberculosis/ | 234,249 |
| #2 | (TB or tuberculo*).mp. [mp=title, abstract, subject headings, heading word, drug trade name, original title, device manufacturer, drug manufacturer, device trade name, keyword] | 319,051 |
| #3 | 1 OR 2 | 321,086 |
| #4 | exp drug therapy/ | 1,946,560 |
| #5 | (treatment or therapy or therapeutic*).mp. [mp=title, abstract, subject headings, heading word, drug trade name, original title, device manufacturer, drug manufacturer, device trade name, keyword] | 7,309,829 |
| #6 | 4 OR 5 | 7,714,699 |
| #7 | exp controlled clinical trial/ | 494,417 |
| #8 | randomi#ed.mp. | 688,909 |
| #9 | trial.mp. | 1,338,760 |
| #10 | 7 OR 8 OR 9 | 1,481,721 |
| **#11** | **3 AND 6 AND 10** | **8,519** |
