# Supplementary material for: What are the most efficacious treatment regimens for isoniazid-resistant tuberculosis? A systematic review and network meta-analysis
Source: Thorax. 2016 Jun 13;71(10):940–9. doi: 10.1136/thoraxjnl-2015-208262 (PMC5036252; doi:10.1136/thoraxjnl-2015-208262)
Supplement: Supplementary file 2 [file thoraxjnl-2015-208262supp_file2.pdf]

SUPPLEMENTARY FILE 2: Fields extracted from studies

Details of the study

- author
- title
- year published
- country of study
- year that study was undertaken
- whether the study focussed on drug resistant patients

Details of participants

- selection criteria
- age
- gender ratio
- site of disease
- HIV status

Microbiology/clinical

- how tuberculosis was diagnosed
- initial drug resistances tested for, including the methodology
- how treatment outcomes and relapses were assessed

Treatment regimens

- drugs
- dose
- length of treatment
- frequency of dosing
- number treated with each regimen

Outcomes

- definition of treatment outcome and how confirmed
- definition of relapse post-treatment and how confirmed
- number of patients with a negative treatment outcome
- number of patients who relapsed and when
- number of patients who died from tuberculosis and when
- number of patients with adverse events, the type of event, and when it happened
- length of follow up post-treatment

- whether doses of treatment were observed
- adherence

Quality assessment

- randomisation
- allocation concealment
- blinding of participants and personnel
- blinding of outcome assessment

If the overall RCT was not focussed on drug resistant disease the risk of bias from selective reporting was deemed high. Attrition thresholds (across the entire study population) were- 10% or greater loss during follow up across all study participants (ignoring exclusions for not fulfilling inclusion criteria), 10% or greater absolute difference in losses between any two study arms.
