# Supplementary material for: What are the most efficacious treatment regimens for isoniazid-resistant tuberculosis? A systematic review and network meta-analysis
Source: Thorax. 2016 Jun 13;71(10):940–9. doi: 10.1136/thoraxjnl-2015-208262 (PMC5036252; doi:10.1136/thoraxjnl-2015-208262)
Supplement: Supplementary file 3 [file thoraxjnl-2015-208262supp_file3.pdf]

SUPPLEMENTARY FILE 3: Pairwise meta-analysis, mixed treatment comparisons approach and publication bias

For the pairwise meta-analysis both Mantel-Haenszel fixed-effects and DerSimonian and Laird random-effects models were used to calculate effect estimates (odds ratios (ORs)) and their associated 95% confidence intervals (CIs). Inconsistency was quantified using the I^2 statistic to determine how much variability in the effect estimates was attributable to heterogeneity.

For the mixed treatment comparisons approach fixed- and random-effects models were fitted, the former assuming common treatment effects across trials and the latter including random-effects for between-trial variation in treatment effects. The random-effect was given a uniform (0,5) prior on the standard deviation scale and within-trial correlation in multi-arm trials corrected for via the adjustment suggested by Welton *et al*.[1] The relative fit of fixed- versus random-effects models was assessed using the deviance information criteria (DIC).[2] Sensitivity analyses were planned to examine the impact of different drug resistance patterns and dose RIF dose (threshold set to be ≥450 mg/day (approximately 10 mg/kg/day)).

Models were implemented in a fully Bayesian framework in WinBUGS,[3] with posterior distributions based on 20,000 samples after a burn-in period of 10,000 iterations. Convergence was assessed by visual examination of parameter chains and the Gelman-Rubin diagnostic.[4] Summary statistics (ORs, relative ranks, absolute differences proportions) and 95% credible intervals (CrIs) were obtained from the posterior distributions produced. Rankings (1=best and n=worst, where n is the number of regimens) should not be seen as a quantification of efficacy, but as an ordering of assessed regimens. The proportion of patients with a negative outcome for the baseline treatment was estimated as the mean expected estimate of events across trials if they had all included an arm with the baseline category.

Inconsistency was assessed in two ways. During the initial stages of model building it was investigated to improve the fit of a fixed-effects model. In the final model pairwise and network meta-analyses OR estimates and their CIs/CrIs were compared to determine if the evidence was systematically inconsistent or simply randomly variable. A more formal node-splitting analysis was not worthwhile given data sparsity.[5]

Network meta-analysis renders publication bias complex to assess because multiple treatment comparisons are considered simultaneously. As network sparsity would have left

more formal tests under-powered to evaluate the likelihood of bias funnel plots and the Harbord test were used on pairwise comparisons only.[6]

References

1 Dias S, Welton NJ, Sutton AJ, et al. NICE DSU Technical Support Document 2: A Generalised Linear Modelling Framework for Pairwise and Network Meta-Analysis of Randomised Controlled Trials. 2014. http://www.nicedsu.org.uk/TSD2%20General%20meta%20analysis%20corrected%2015April2014.pdf (accessed Apr 2014).

2 Spiegelhalter DJ, Best NG, Carlin BP, et al. Bayesian measures of model complexity and fit. *J R Stat Soc Series B Stat Methodol* 2002;64:583-640.

3 Spiegelhalter DJ, Best NG. Bayesian approaches to multiple sources of evidence and uncertainty in complex cost-effectiveness modelling. *Stat Med* 2003;22:3687-709.

4 Brooks SP. MCMC Convergence Diagnosis via Multivariate Bounds on Log-Concave Densities. *Ann Stat* 1998;26:398-433.

5 Dias S, Welton NJ, Caldwell DM, et al. Checking consistency in mixed treatment comparison meta-analysis. *Stat Med* 2010;29:932-44.

6 Harbord RM, Harris RJ, Sterne JAC. Updated tests for small-study effects in meta-analyses. *Stata J* 2009;9:197-210.
