# Supplementary material for: What are the most efficacious treatment regimens for isoniazid-resistant tuberculosis? A systematic review and network meta-analysis
Source: Thorax. 2016 Jun 13;71(10):940–9. doi: 10.1136/thoraxjnl-2015-208262 (PMC5036252; doi:10.1136/thoraxjnl-2015-208262)

SUPPLEMENTARY FILE 4: Flow chart of selection

Flow chart of inclusion within the systematic review. At full text extraction stage 'not randomised' relates to the functionality of the randomisation for DR patients (randomisation may have been broken). DR- drug resistance, INH- isoniazid, MDR- multidrug resistant, R- resistant, RCT- randomised controlled trial, STM- streptomycin, TB- tuberculosis, WoS- Web of Science, XDR- extensively drug resistant

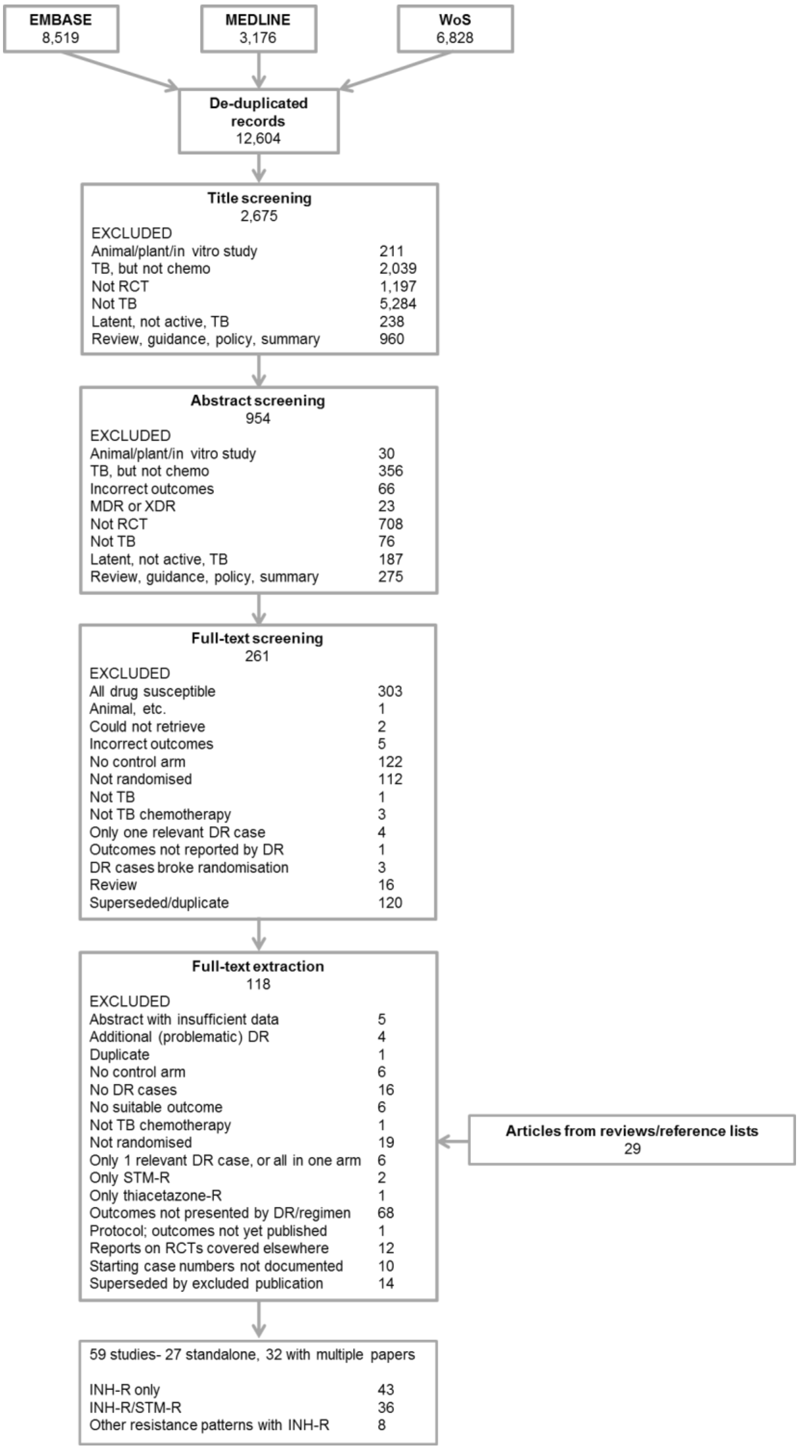

Supplement: Supplementary file 4 [file thoraxjnl-2015-208262supp_file4.pdf]
