# Supplementary material for: What are the most efficacious treatment regimens for isoniazid-resistant tuberculosis? A systematic review and network meta-analysis
Source: Thorax. 2016 Jun 13;71(10):940–9. doi: 10.1136/thoraxjnl-2015-208262 (PMC5036252; doi:10.1136/thoraxjnl-2015-208262)
Supplement: Supplementary file 5 [file thoraxjnl-2015-208262supp_file5.pdf]

SUPPLEMENTARY FILE 5: Studies excluded at the full text extraction stage

DR- drug resistance, INH-R- isoniazid resistant, STM-R- streptomycin resistant, RCT- randomised controlled trail

Author	Reason for exclusion
Abdali[1]	Abstract with insufficient data, cannot find subsequent publication
Aber[2]	Reports on RCTs covered elsewhere, not original study report
Ait-Khaled[3]	Starting case numbers not documented
Aluoch[4]	Reports on RCTs covered elsewhere, not original study report
Aluoch[5]	Reports on RCTs covered elsewhere, not original study report
Anastasatu[6]	No DR cases
Angel[7]	Reports on RCTs covered elsewhere, not original study report
Angel[8]	No outcomes for DR cases or by regimen
Angel[9]	Only one INH-R, or all INH-R in single arm
Aquinas[10]	No suitable outcome
Auer[11]	No outcomes for DR cases or by regimen
Aung[12]	Starting case numbers not documented
Aziz[13]	(Extra) DR issues
Baba[14]	No outcomes for DR cases or by regimen
Baba[15]	Superseded by publication otherwise excluded
Balasubramanian[16]	No outcomes for DR cases or by regimen
Bignall[17]	Superseded by publication otherwise excluded
Bignall[18]	Superseded by publication otherwise excluded
Bignall[19]	No outcomes for DR cases or by regimen
Bignall[20]	Not randomised
Bignall[21]	Superseded by publication otherwise excluded
Boszorme[22]	No outcomes for DR cases or by regimen
Boszormeny[23]	No outcomes for DR cases or by regimen
Bozeman[24]	Only one INH-R, or all INH-R in single arm
Brandli[25]	Starting case numbers not documented
Brandli[26]	No outcomes for DR cases or by regimen
British Medical Research Council[27]	Only one INH-R, or all INH-R in single arm
British Thoracic Association[28]	Only STM-R
British Thoracic Society and Tuberculosis Association[29]	No outcomes for DR cases or by regimen
British Thoracic Society Research Committee[30]	No outcomes for DR cases or by regimen
Burman[31]	No outcomes for DR cases or by regimen
Campbell[32]	No outcomes for DR cases or by regimen
Campbell[33]	Superseded by publication otherwise excluded
Castelo[34]	No outcomes for DR cases or by regimen
Chan[35]	Reports on RCTs covered elsewhere, not original study report
Cheung-Yam[36]	Reports on RCTs covered elsewhere, not original study report
Chicou[37]	Not randomised

Author	Reason for exclusion
Chicou[38]	Starting case numbers not documented
Chiolero[39]	Starting case numbers not documented
Chmelev[40]	No outcomes for DR cases or by regimen
Chukanov[41]	No outcomes for DR cases or by regimen
Citron[42]	Only STM-R
Conde[43]	No outcomes for DR cases or by regimen
Cooperative Study Unit on Chemotherapy of Tuberculosis of the National Sanatoria [44]	No DR cases
Cooperative Study Unit on Chemotherapy of Tuberculosis of the National Sanatoria in Japan[45]	No DR cases
Cooperative Study Unit on Chemotherapy of Tuberculosis of the National Sanatoria in Japan[46]	No DR cases
Cooperative Study Unit on Chemotherapy of Tuberculosis of the National Sanatoria in Japan[47]	No DR cases
Cooperative Study Unit on Chemotherapy of Tuberculosis of the National Sanatoria in Japan[48]	No outcomes for DR cases or by regimen
Cowie[49]	Not randomised
de Figueiredo[50]	Not randomised
Demidik[51]	Not chemotherapy
Diacon[52]	No DR cases
Dingley[53]	No outcomes for DR cases or by regimen
Donomae[54]	(Extra) DR issues
Dormer[55]	No DR cases
Doster[56]	Not randomised
Doster[57]	No DR cases
East African/British Medical Research Council[58]	Not randomised
El Fassi el Fahr[59]	Not randomised
Enta and Eec-tuberculosis Concerted Actions[60]	Abstract with insufficient data, cannot find subsequent publication
Eule[61]	No DR cases
Farga[62]	No outcomes for DR cases or by regimen
Favez[63]	No DR cases
Favez[64]	Starting case numbers not documented
Favez[65]	No DR cases
Felten[66]	No outcomes for DR cases or by regimen
Gillespie[67]	No outcomes for DR cases or by regimen
Girling[68]	Not randomised
Gomi[69]	Not randomised
Gomi[70]	Not randomised
Gonzales Montanler[71]	Superseded by publication otherwise excluded
Gonzalez-Montaner[72]	No outcomes for DR cases or by regimen
Gonzalez-Montaner[73]	No DR cases
Grassi[74]	No DR cases

Author	Reason for exclusion
Gupta[75]	Superseded by publication otherwise excluded
Gyselen[76]	Only one INH-R, or all INH-R in single arm
He[77]	Starting case numbers not documented
Heemskerk[78]	Protocol; outcomes not yet published
Holden[79]	No control arm
Hong Kong Chest Service/British Medical Research Council[80]	(Extra) DR issues
Hong Kong Tuberculosis Treat Service/British Medical Research Council[81]	No outcomes for DR cases or by regimen
Hong Kong Tuberculosis Treat Service/British Medical Research Council[82]	Not randomised
Hong Kong Tuberculosis Treat Service/British Medical Research Council[83]	No suitable outcome
Hong Kong Tuberculosis Treat Service/Brompton Hospital/British Medical Research Council[84]	Not randomised
Hong Kong Tuberculosis Treat Service/Brompton Hospital/British Medical Research Council[85]	Not randomised
Hong Kong Tuberculosis Treatment Services/British Medical Research Council[86]	Not randomised
Huang[87]	No outcomes for DR cases or by regimen
Huet[88]	Only thiacetazone resistant
International Union against Tuberculosis [89]	Only one INH-R, or all INH-R in single arm
Jawahar[90]	No outcomes for DR cases or by regimen
Kang[91]	Starting case numbers not documented
Kim[92]	No DR cases
Kleeberg[93]	No outcomes for DR cases or by regimen
Larbaoui[94]	Superseded by publication otherwise excluded
Larbaoui[95]	Some sensitive cases, quite imbalanced by arm
Larbaoui[96]	No outcomes for DR cases or by regimen
Li[97]	Starting case numbers not documented
Lienhardt[98]	No outcomes for DR cases or by regimen
Lind[99]	Superseded by publication otherwise excluded
Long[100]	No outcomes for DR cases or by regimen
Macnab[101]	No outcomes for DR cases or by regimen
Maekawa[102]	Abstract with insufficient data, cannot find subsequent publication
Mawer[103]	No outcomes for DR cases or by regimen
Medical Research Council Working Party on Tuberculosis of the Spine[104]	No outcomes for DR cases or by regimen
Meissner[105]	Only one INH-R, or all INH-R in single arm
Merle[106]	No outcomes for DR cases or by regimen
MRC Working Party on Tuberculosis of the Spine[107]	Reports on RCTs covered elsewhere, not original study report
Mukadi[108]	Superseded by publication otherwise excluded
Narayanan[109]	Reports on RCTs covered elsewhere, not original study report
National Cooperative Group On Clinical Study Of Rifapentine[110]	No outcomes for DR cases or by regimen

Author	Reason for exclusion
Nitti[111]	(Extra) DR issues
Nunn[112]	No outcomes for DR cases or by regimen
Pamra[113]	No outcomes for DR cases or by regimen
Perriens[114]	No outcomes for DR cases or by regimen
Pilheu[115]	No outcomes for DR cases or by regimen
Poh[116]	Reports on RCTs covered elsewhere, not original study report
Pult[117]	No control arm
Punnotok[118]	No outcomes for DR cases or by regimen
Rabier[119]	(Extra) DR issues
Radenbach[120]	No outcomes for DR cases or by regimen
Ramasamy[121]	No outcomes for DR cases or by regimen
Research Committee of the British Tuberculosis Association[122]	Not randomised
Research Committee of the Tuberculosis Association of India [123]	No outcomes for DR cases or by regimen
Reves[124]	No control arm
Rey[125]	No outcomes for DR cases or by regimen
Rist[126]	No outcomes for DR cases or by regimen
Rist[127]	Not randomised
Robitzek[128]	Superseded by publication otherwise excluded
Rogowski[129]	Abstract with insufficient data, cannot find subsequent publication
Ruslami[130]	No outcomes for DR cases or by regimen
Salinger[131]	No outcomes for DR cases or by regimen
Schutz[132]	No outcomes for DR cases or by regimen
Schutz[133]	No suitable outcome
Shennan[134]	No DR cases
Shishito[135]	No DR cases
Snider[136]	Reports on RCTs covered elsewhere, not original study report
Snider[137]	No outcomes for DR cases or by regimen
Sokolova[138]	No control arm
Sokolova[139]	No control arm
Sokolova[140]	No outcomes for DR cases or by regimen
Somner[141]	Reports on RCTs covered elsewhere, not original study report
Somner[142]	Superseded by publication otherwise excluded
Stahle[143]	No outcomes for DR cases or by regimen
Steininger[144]	No outcomes for DR cases or by regimen
Sung[145]	No outcomes for DR cases or by regimen
Sutherland[146]	No outcomes for DR cases or by regimen
Swai[147]	Reports on RCTs covered elsewhere, not original study report
Tala[148]	Abstract with insufficient data, cannot find subsequent publication
The British Thoracic Society Research Committee[149]	No outcomes for DR cases or by regimen

Author	Reason for exclusion
Thwaites[150]	No outcomes for DR cases or by regimen
Tripathy[151]	No outcomes for DR cases or by regimen
Tripathy[152]	Not randomised
Tuberculosis Association of India[153]	No outcomes for DR cases or by regimen
Tuberculosis Chemotherapy Centre, Madras[154]	No outcomes for DR cases or by regimen
United States Public Health Service Tuberculosis Therapy Trial[155]	No outcomes for DR cases or by regimen
Valenzuela[156]	No outcomes for DR cases or by regimen
Velayutham[157]	No suitable outcome
Viswanat[158]	No outcomes for DR cases or by regimen
Viswanathan[159]	No outcomes for DR cases or by regimen
Viswanathan[160]	No control arm
Wang[161]	No suitable outcome
Wissenschaftliche Arbeitsgemeinschaft für die Therapie von Lungenkrankheiten[162]	No suitable outcome
Yamamoto[163]	No outcomes for DR cases or by regimen
Yan[164]	Superseded by publication otherwise excluded
Yan[165]	Superseded by publication otherwise excluded
Yan[166]	Starting case numbers not documented
Yang[167]	Not randomised
Young[168]	No outcomes for DR cases or by regimen
Youssef[169]	No outcomes for DR cases or by regimen
Yu[170]	No outcomes for DR cases or by regimen
Zhang[171]	No outcomes for DR cases or by regimen

Reference list

1 Abdali OE, Khair OA, Kamal MF, et al. The clinical efficacy, patient adherence to treatment and side effects of two 6 months regimes for the treatment of pulmonary TB. *Ann Thorac Med* 2009;4:01.

2 Aber VR, Nunn AJ. Short term chemotherapy of tuberculosis. Factors affecting relapse following short term chemotherapy. *Bull Int Union Tuberc* 1978;53:276-80.

3 Ait-Khaled N, Benadjila H, Loucif MS, et al. Controlled therapeutic trial on three second-line chemotherapy regimens (one of short duration) in pulmonary tuberculosis. *Bull Int Union Tuberc* 1976;51:95-102.

4 Aluoch JA. Controlled Clinical-Trial of 4 Short-Course Regimens of Chemotherapy for 2 Durations in the Treatment of Pulmonary Tuberculosis. *East Afr Med J* 1979;56:143.

5 Aluoch JA. 4th study of short term chemotherapy by the African and British Medical Research Councils. *Bull Int Union Tuberc* 1978;53:256-7.

6 Anastasatu C, Bercea O, Colan E. Controlled Clinical Trial on Smear Negative X-Ray Positive New Cases with A View to Establishing If and How to Treat Them. *Bull Int Union Tuberc* 1982;57:89.

7 ANGEL JH. Bta Short-Course Chemotherapy Studies. *Chest* 1981;80:722-4.

8 ANGEL JH, Campbell IA, Dyson A, et al. Short Course Chemo Therapy in Pulmonary Tuberculosis A Controlled Trial by the British Thoracic and Tuberculosis Association. *Lancet* 1976;2:1102-4.

9 ANGEL JH, Bhatia AL, Devadatta S, et al. A controlled comparison of cycloserine plus ethionamide with cycloserine plus thiacetazone in patients with active pulmonary tuberculosis despite prolonged previous chemotherapy. *Tubercle* 1963;44:215-24.
10 Aquinas M, Allan WG, Horsfall PA, et al. Adverse reactions to daily and intermittent rifampicin regimens for pulmonary tuberculosis in Hong Kong. *Br Med J* 1972;1:765-71.
11 Auer C, Montejo L, Lagahid J, et al. Randomized controlled trial of daily vs thrice-weekly anti-TB therapy in public health centres of Manila, Philippines. *Int J Tuberc Lung Dis* 2003;7:147.
12 Aung KJM, Declercq E, Ali M, et al. Extension of the intensive phase reduces relapse but not failure in a regimen with rifampicin throughout. *Int J Tuberc Lung Dis* 2012;16:01.
13 Aziz A, Ishaq M, Jaffer NA, et al. Clinical-Trial of 2 Short-Course (6-Month) Regimens and A Standard Regimen (12-Month) Chemotherapy in Retreatment of Pulmonary Tuberculosis in Pakistan - Results 18 Months After Completion of Treatment (Lahore Tuberculosis Study). *Am Rev Respir Dis* 1986;134:1056-61.
14 Baba H, Shinkai A, Izuchi R, et al. Long-Term Results of Short Course Chemotherapy for Pulmonary Tuberculosis Second Study B Results at 6 Years After the End of 4-9 Months of Chemotherapy for Pulmonary Tuberculosis. *Kekkaku* 1987;62:511-20.
15 Baba H, Shinkai A, Izuchi R, et al. Controlled Clinical Trial of 4 6 Month Regimens of Chemo Therapy for Pulmonary Tuberculosis. *Bull Int Union Tuberc* 1982;57:20.
16 Balasubramanian R, Nagarajan M, Balambal R, et al. Randomised controlled clinical trial of short course chemotherapy in abdominal tuberculosis: a five-year report. *Int J Tuberc Lung Dis* 1997;1:44-51.
17 Bignall JR. 3rd Chemotherapy Trial. *Bull Int Union Tuberc* 1968;40:48-9.
18 Bignall JR. Report on the present state of the second chemotherapy trial. *Bull Int Union Tuberc* 1967;39:91-6.
19 Bignall JR. A Controlled Trial of 5 Intermittent Chemo Therapy Regimens in Pulmonary Tuberculosis the 4th International Trial of the International Unit Against Tuberculosis. *Bull Int Union Tuberc* 1974;49:392-5.
20 Bignall JR. A controlled trial of three regimens of self-administered and supervised chemotherapy for pulmonary tuberculosis. A. General report. *Bull Int Union Tuberc* 1970;44:8-37.
21 Bignall JR. A comparison of regimens of ethionamide, pyrazinamide and cycloserine in re-treatment of patients with pulmonary tuberculosis. *Bull Int Union Tuberc* 1968;41:175-7.
22 Boszorme M, Schweige O. A Controlled Clinical Trial of Drug Treatment of New Cases of Pulmonary Tuberculosis. *Scand J Respir Dis* 1968;S:189-&.
23 Boszormenyi M, Fauszt I, Barat I, et al. A controlled clinical trial of ethionamide, cycloserine and pyrazinamide in previously treated patients with pulmonary tuberculosis. *Tubercle* 1965;46:143-50.
24 Bozeman L, Burman W, Metchock B, et al. Fluoroquinolone susceptibility among Mycobacterium tuberculosis isolates from the United States and Canada. *Clin Infect Dis* 2005;40:386-91.
25 Brandli O, Haegi V, Villiger B, et al. Short-term therapy of lung tuberculosis using a fixed combination of isoniazid, rifampicin and pyrazinamide. Results after 2 years. *Schweiz Med Wochenschr* 1989;119:299-305.
26 Brandli O, Dreher D, Morger D. Results of short-term tuberculosis therapy with isoniazid, rifampicin and pyrazinamide. *Schweiz Med Wochenschr* 1993;123:1300-6.
27 British Medical Research Council. Co-operative controlled trial of a standard regimen of streptomycin, PAS and isoniazid and three alternative regimens of chemotherapy in Britain. A report from the British Medical Research Council. *Tubercle* 1973;54:99-129.

28 British Thoracic Association. A controlled trial of six months chemotherapy in pulmonary tuberculosis. Second report: results during the 24 months after the end of chemotherapy. British Thoracic Association. *The American review of respiratory disease* 1982;126:460-2.
29 British Thoracic Society and Tuberculosis Association. Short-course chemotherapy in pulmonary tuberculosis. A controlled trial by the British Thoracic and Tuberculosis Association. *Lancet* 1975;1:119-24.
30 British Thoracic Society Research Committee. Short course chemotherapy for tuberculosis of lymph nodes: a controlled trial. British Thoracic Society Research Committee. *British Medical Journal Clinical Research Ed* 1985;290:1106-8.
31 Burman WJ, Goldberg S, Johnson JL, et al. Moxifloxacin versus ethambutol in the first 2 months of treatment for pulmonary tuberculosis. *Am J Respir Crit Care Med* 2006;174:331-8.
32 Campbell IA, Ormerod LP, Friend JAR, et al. 6-Months Versus 9-Months Chemotherapy for Tuberculosis of Lymph-Nodes - Preliminary-Results. *Respir Med* 1992;86:15-9.
33 Campbell IA. 6 Months Chemotherapy in Pulmonary Tuberculosis - A Controlled Trial by the British-Thoracic-Association. *Br J Dis Chest* 1980;74:415.
34 Castelo A, Jardim JRB, Goihman S, et al. Comparison of Daily and Twice-Weekly Regimens to Treat Pulmonary Tuberculosis. *Lancet* 1989;2:1173-6.
35 Chan SL. Hong-Kong-Chest-Service - British-Medical-Research-Council Controlled Trial of 4 3-Times-Weekly Regimens and A Daily Regimen All Given for 6 Months for Pulmonary Tuberculosis. *Thorax* 1981;36:221.
36 Cheung-Yam LYC. Smear-negative pulmonary tuberculosis: Controlled trial of 3-month and 2-month regimens. *Bull Int Union Tuberc* 1979;54:25.
37 Chicou J, Hetrick G, Le Hir M, et al. Trial chemotherapy of 103 cases of pulmonary tuberculosis, all resistant to 1 or several antibiotics, in a country with high tuberculosis endemia (early results after at least 4 months' treatment. Reflections and practical suggestions on so-called "chronic" tuberculosis). *Rev Tuberc Pneumol (Paris)* 1962;26:867-83.
38 Chicou FJ, Hetrick G, Huet M, et al. Comparative Assay of 2 Types of Oral Treatment of Pulmonary Tuberculosis (Ethionamide+Isoniazide Thioacetazone+Isoniazide). *Bull World Health Organ* 1968;39:731.
39 Chiolero R. Rifampicin in the treatment of active pulmonary tuberculosis. Controlled trial of isoniazid-rifampicin-thiocarlide versus isoniazid-streptomycin-thiocarlide in untreated tuberculosis. Treatment of relapses with the combination rifampicin-ethambutol-thiocarlide. *Schweiz Med Wochenschr* 1974;104:356-64.
40 Chmelev NA, Alyabina MG. Collective studies from the whole of the Soviet Union on controlled chemotherapy of pulmonary tuberculosis. *Bull Int Union Tuberc* 1972;47:22-9.
41 Chukanov VI, Saatsazova ES, Fedotova NN. Effectiveness of various chemotherapy schedules in patients with newly detected limited nondestructive pulmonary tuberculosis. *Probl Tuberk* 1988;57-60.
42 Citron KM. A Controlled Trial of 6 Months Chemo Therapy in Pulmonary Tuberculosis 1. Results During Chemo Therapy. *Br J Dis Chest* 1981;75:141-53.
43 Conde MB, Cavalcante SC, Dalcolmo M, et al. A Phase 2 Trial of a Rifapentine Plus Moxifloxacin-Based Regimen for Pulmonary TB Treatment. *Top Antivir Med* 2014;22:47.
44 Clinical comparison of capreomycin and kanamycin, with special reference to the effects of CPM in the KM-resistant cases. *Saishin igaku* 1968;23:2400-4.
45 Cooperative Study Unit on Chemotherapy of Tuberculosis of the National Sanatoria in Japan. Evaluation of alternative regimen of SM-INH-PAS and KM-TH-CS at three months interval in initial treatment of far advanced pulmonary tuberculosis. A report of the 12th series controlled trial of chemotherapy. *Kekkaku* 1972;47:151-8.

46 Cooperative Study Unit on Chemotherapy of Tuberculosis of the National Sanatoria in Japan. Comparison of three regimens, INH-SM-PAS, INH-SM-RFP and INH-PAS-RFP, in the original treatment cases of pulmonary tuberculosis. Report of the 13th series study (original treatment study). *Kekkaku* 1973;48:235-45.

47 Cooperative Study Unit on Chemotherapy of Tuberculosis of the National Sanatoria in Japan. Comparison of 1.0g streptomycin, 0.5g streptomycin and 1.0g capreomycin three times a week combined with PAS and isoniazid in original treatment for pulmonary tuberculosis. Report of the 17th series of controlled trial of chemotherapy. *Kekkaku* 1976;51:271-8.

48 Cooperative Study Unit on Chemotherapy of Tuberculosis of the National Sanatoria in Japan. Clinical efficacy of new anti-tuberculosis drugs. Ethionamide, cycloserine and dextro-2-2'-(ethylenediimino)-di-1-butanol (ethambutol, ebutol) in re-treatment: Eighth series of controlled trials, II. The cooperative study unit on chemotherapy of tuberculosis of the national sanatoria in Japan. *Tubercle* 1966;47:1966.

49 Cowie RL, Brink BA. Short-course chemotherapy for pulmonary tuberculosis with a rifampicin-isoniazid-pyrazinamide combination tablet. *S Afr Med J* 1990;77:390-1.

50 De Figueiredo FP, Alves BA, Laborne Valle JH, et al. Short Duration Chemo Therapy of Pulmonary Tuberculosis A Pilot Trial. *Bull Int Union Tuberc* 1974;49:382-8.

51 Demidik SN, Sukhanov DS. Cycloferon in the complex therapy of patients with widespread forms of pulmonary tuberculosis. *Eksp Klin Farmakol* 2012;75:17-20.

52 Diacon A, Dawson R, Van Niekerk C, et al. Phase 2 Trial of a Novel 3-Drug Regimen for Both MDR and Drug Sensitive (DS) TB. *Abstr Intersci Conf Antimicrob Agents Chemother Intersci Conf Antimicrob Agents Chemother* 2011;51:P-931b.

53 Dingley HB, Sehgal KL. Treatment of Pulmonary Tuberculosis in Children - Controlled Study. *Indian Pediatr* 1974;11:289-95.

54 Donomae I, Fujita S, Gomi J, et al. Clinical Evaluation of Rifampicin for Pulmonary Tuberculosis Part 3 A Controlled Trial on the Clinical Effects of Chemo Therapy with Rifampicin Ethambutol and Viomycin Ethambutol in Re Treatment of Cases of Pulmonary Tuberculosis. *Kekkaku* 1971;46:371-7.

55 Dormer BA. Comparative controlled trial of rimactane in pulmonary tuberculosis. *Int Z Klin Pharmakol Ther Toxikol* 1969;2:188.

56 Doster B, Newman R. Ethambutol Anti Infect in Re Treatment of Pulmonary Tuberculosis Usa Public Health Service Tuberculosis Therapy Trial Human Isoniazid Cyclo Serine Capreomycin Streptomycin Kanamycin Viomycin Ethionamide Anti Infect. *Am Rev Respir Dis* 1968;98:825-36.

57 Doster B, Murray FJ, Newman R, et al. Ethambutol in Initial Treatment of Pulmonary Tuberculosis - Us Public-Health Service Tuberculosis Therapy Trials. *Am Rev Respir Dis* 1973;107:177-90.

58 East African/British Medical Research Council. Comparative Trial of Isoniazid Alone in Low and High Dosage and Isoniazid Plus Pas in the Treatment of Acute Pulmonary Tuberculosis in East Africans. *Tubercle* 1960;41:83-102.

59 El Fassi El FM, Ivanovic M, Lahlou M. A trial of essentially ambulatory and intermittent treatment of patients with tb of the lung from drug-resistant M. tuberculosis. *Journal de Medecine du Maroc* 1968;4:8-9.

60 Enta And Eec-tuberculosis Concerted Actions. Tuberculosis (TBC) in HIV-infected patients (P): A multicentric, randomized comparative study of a three versus a four drug regimen: European Tuberculosis Study Group. *Int Conf AIDS* 1993;339.

61 Eule H, Iwainsky H, Kaernbach E, et al. Intermittent treatment using isoniazid and rifampicin once a week: final results of a controlled trial. *Bull Int Union Tuberc* 1976;51:115-9.

62 Farga V, Valenzuela P, Mendoza F, et al. Short-term chemotherapy. Controlled clinical trials in Chile. *Bull Int Union Tuberc* 1983;58:102-7.

63 Favez G, Leuenberger P. A Controlled Trial of Individually-Adapted Short-Course Chemotherapy Versus 2-Year Scheme in Original Treatment of Pulmonary Tuberculosis - Report After A 5-Year Follow-Up. *Chest* 1982;82:426-9.

64 Favez G, Maillard JM, Vouilloz M. Comparative results of medium and increased doses of isoniazid in the triple treatment of overt pulmonary tuberculosis. *Schweiz Med Wochenschr* 1968;98:1392-5.
65 Favez G, Gonin JD, Guberan E, et al. Comparative results of treatment of pulmonary tuberculosis with the compounds of benzoyl-PAS-Ca-isoniazide and streptomycin-isoniazid. *Schweiz Med Wochenschr* 1969;99:150-6.
66 Felten MK. Importance of rifampicin in combined daily/intermittent chemotherapy for tuberculosis. *S Afr Med J* 1989;75:524-6.
67 Gillespie SH, Crook AM, McHugh TD, et al. Four-month moxifloxacin-based regimens for drug-sensitive tuberculosis. *N Engl J Med* 2014;371:23.
68 Girling DJ. A controlled clinical comparison of 6 and 8 months of antituberculosis chemotherapy in the treatment of patients with silicotuberculosis in Hong Kong. *Am Rev Respir Dis* 1991;143:262-7.
69 Gomi J, Aoyagi T, Fukuhara Y, et al. The short course therapies by Ryoken's method. 9 month and 12 month regimens including rifampicin (RFP) plus isoniazid (INH) in bacteriologically proved pulmonary tuberculosis. *Kekkaku* 1985;60:435-45.
70 Gomi J. The treatment of pulmonary tuberculosis with kanamycin. *Chemotherapia (Basel)* 1963;6:35-41.
71 Gonzales Montanler J, Shoenfelder J, Dolfi L, et al. Rifabutin-containing regimens for pulmonary tuberculosis: early assessment of treatment effectiveness. *Tuber Lung Dis* 1996;77:100.
72 Gonzalez-Montaner LJ, Natal S, Yongchaiyud P, et al. Rifabutin for the treatment of newly-diagnosed pulmonary tuberculosis: a multinational, randomized, comparative study versus Rifampicin. Rifabutin Study Group. *Tuber Lung Dis* 1994;75:341-7.
73 Gonzalez Montaner LJ, Palma BO, Abbate E, et al. A comparison of the therapeutic efficacy of two drug combinations in cases of previously untreated open tuberculosis. *Prax Klin Pneumol* 1978;32:717-20.
74 Grassi C. Treatment of chronic tuberculosis with ethambutol and prothionamide associated with rifomycin of capreomycin. *Acta Tuberc Pneumol Belg* 1969;60:450-4.
75 Gupta ML, Bhattacharya P. Rifabutin for the treatment of newly diagnosed pulmonary tuberculosis: A multinational randomized comparative study versus rifampicin. *Tuber Lung Dis* 1995;76:582.
76 Gyselen A, Verbist L, Cosemans J, et al. A cooperative study on rifampicin in original treatment of advanced pulmonary tuberculosis. *Acta Tuberc Pneumol Belg* 1969;60:563-76.
77 He GJ. [A comparative study of rifapentine treatment and three years follow-up on initial pulmonary tuberculous]. [Chinese]. *Chung-Hua Chieh Ho Ho Hu Hsi Tsa Chih Chinese Journal of Tuberculosis & Respiratory Diseases* 122;16:73-6.
78 Heemskerk D, Day J, Chau TT, et al. Intensified treatment with high dose rifampicin and levofloxacin compared to standard treatment for adult patients with tuberculous meningitis (TBM-IT): protocol for a randomized controlled trial. *Trials* 2011;12:25.
79 Holden H, Shane SJ, Vanrooyen CE. Ristocetin Treatment Failure in Pulmonary Tuberculosis. *Am Rev Respir Dis* 1960;82:736-7.
80 Hong Kong Chest Service/British Medical Research Council. A controlled study of rifabutin and an uncontrolled study of ofloxacin in the retreatment of patients with pulmonary tuberculosis resistant to isoniazid, streptomycin and rifampicin. *Tuber Lung Dis* 1992;73:59-67.
81 Hong Kong Tuberc Treat Serv, Br Med Res COUN. The Influence of Age and Sex on the Incidence of the Flu Syndrome and Rifampicin Dependent Antibodies in Patients on Intermittent Rifampicin for Tuberculosis. *Tubercle* 1975;56:173-8.
82 Hong Kong Tuberculosis Treat Service/British Medical Research Council. A study in Hong Kong to evaluate the role of pretreatment susceptibility tests in the selection of regimens of chemotherapy for pulmonary tuberculosis--second report. A Hong Kong Tuberculosis Treatment Services/British Medical Research Council Investigation. *Tubercle* 1974;55:169-92.

83 Hong Kong Tuberculosis Treat Service/British Medical Research Council. A controlled clinical trial of small daily doses of rifampicin in the prevention of adverse reactions to the drug in a once-weekly regimen of chemotherapy. A Hong Kong Tuberculosis Treatment Services-British Medical Research Council Investigation. *Clin Allergy* 1974;4:1-12.
84 Hong Kong Tuberc Treat Serv Brompton Hosp Br Med Res Counc. A Controlled Trial of Daily and Intermittent Rifampicin Plus Ethambutol in the Re Treatment of Patients with Pulmonary Tuberculosis Results Up to 30 Months. *Tubercle* 1975;56:179-89.
85 Hong Kong Tuberc Treat Serv, Brompton Hosp E, Brit Med Res Counc Invest. A Controlled Clinical Trial of Daily and Intermittent Regimens of Rifampicin Plus Ethambutol in the Re Treatment of Patients with Pulmonary Tuberculosis in Hong-Kong. *Tubercle* 1974;55:1-27.
86 Hong Kong Tuberculosis Treatment Services/British Medical Research Council. A controlled clinical trial of small daily doses of rifampicin in the prevention of adverse reactions to the drug in a once weekly regimen of chemotherapy in Hong Kong: Second report: the results at 12 months. *Tubercle* 1974;55:193-210.
87 Huang C-S, Wu C-C. Observation of the clinical efficacy of sparfloxacin in the treatment of multiple drug resistance pneumonial tuberculosis. *Zhongguo Kang Sheng Su Za Zhi* 2000;25:302-3.
88 Huet M, Boube G, Rist N. Trial of therapy of pulmonary tuberculosis in Cameroon with a combination of isoniazid and thiacetazone. Survey of results after 6 months. *Arch Inst Pasteur Tunis* 1973;50:267-90.
89 International Union against Tuberculosis. An international investigation of the efficacy of chemotherapy in previously untreated patients with pulmonary tuberculosis. A trial directed by the committee on treatment and the committee on bacteriology and immunology of the International Union against Tuberculosis. *Bull Int Union Tuberc* 1964;34:82-191.
90 Jawahar MS, Banurekha VV, Paramasivan CN, et al. Randomized Clinical Trial of Thrice-Weekly 4-Month Moxifloxacin or Gatifloxacin Containing Regimens in the Treatment of New Sputum Positive Pulmonary Tuberculosis Patients. *Plos One* 2013;8:e67030.
91 Kang WL, Xie YG, Tan WG, et al. Study on the efficacy and safety of short-term treatment including fluoroquinolones anti-tuberculosis drugs for rifampicin resistant pulmonary tuberculosis. *Zhong Guo Di Fang Bing Xue Za Zhi* 2009;30:179-83.
92 Kim M, Park D, Sohn J, et al. A prospective randomized, controlled study to determine the optimal duration of antituberculous therapy for tuberculous cervical lymphadenitis. *Abstr Intersci Conf Antimicrob Agents Chemother Intersci Conf Antimicrob Agents Chemother* 2003;43:416.
93 Kleeberg HH. Pulmonary Tuberculosis Treated with Isoprodian and Rifampicin Or Pyrazinamide. *Chemotherapy (Los Angel)* 1987;33:219-28.
94 Larbaoui D, Lamrani Z, Boulahbal F. Final results of a controlled chemotherapy trial comparing four 'heavy' regimens of short-course chemotherapy in pulmonary tuberculosis. *Bull Int Union Tuberc* 1982;57:20.
95 Larbaoui D, Alloula R, Boulahbal F. Comparison between four reserve treatments using rifampicin and ethambutol: three long intermittent treatments and one short daily one. *Rev Fr Mal Respir* 1977;5:421-30.
96 Larbaoui D, Lamrani Z, Boulahbal F. Final results of a controlled trial comparing four heavy short-course chemotherapy regimens in the treatment of pulmonary tuberculosis. *Bull Int Union Tuberc* 1984;59:30-3.
97 Li L, Zheng SH, Chu NH, et al. Effects of two treatment regimens for drug-resistant tuberculosis in tuberculosis control project areas: a comparative study. *Zhonghua Yi Xue Za Zhi (Taipei)* 2008;88:3387-91.
98 Lienhardt C, Cook SV, Burgos M, et al. Efficacy and Safety of a 4-Drug Fixed-Dose Combination Regimen Compared With Separate Drugs for Treatment of Pulmonary Tuberculosis The Study C Randomized Controlled Trial. *JAMA* 2011;305:1415-23.

99 Lind A, Rist N. Results of the second chemotherapy trial of the IUAT. Bacteriological part. *Bull Int Union Tuberc* 1968;41:178.
100 Long MW, Snider DE, Jr., Farer LS. U.S. Public Health Service Cooperative trial of three rifampin-isoniazid regimens in treatment of pulmonary tuberculosis. *Am Rev Respir Dis* 1979;119:879-94.
101 Macnab MF, Bohmer PD, Seager JR. Evaluation of the 3-drug combination, Rifater, versus 4-drug therapy in the ambulatory treatment of tuberculosis in Cape Town. *S Afr Med J* 1994;84:325-8.
102 Proceedings: Chemotherapy in tuberculosis. (3) Side effects. *Kekkaku* 1975;50:76-7.
103 Mawer C, Ignatenko NV, Wares DF, et al. Comparison of the effectiveness of WHO short-course chemotherapy and standard Russian antituberculous regimens in Tomsk, western Siberia. *Lancet* 2001;358:11.
104 Med Res Counc Work Party Tuberc Spine (Uk). A Controlled Trial of Six-Month and Nine-Month Regimens of Chemotherapy in Patients Undergoing Radical Surgery for Tuberculosis of the Spine in Hong Kong. *Tubercle* 1986;67:243-60.
105 Meissner G. Rifampicin in newly detected, untreated cases of pulmonary tuberculosis. *Acta Tuberc Pneumol Belg* 1969;60:554-6.
106 Merle CS, Fielding K, Sow OB, et al. A four-month gatifloxacin-containing regimen for treating tuberculosis. *N Engl J Med* 2014;371:23.
107 MRC Working Party on Tuberculosis of the Spine. Five-year assessment of controlled trials of short-course chemotherapy regimens of 6, 9 or 18 months' duration for spinal tuberculosis in patients ambulatory from the start or undergoing radical surgery. Fourteenth report of the Medical Research Council Working Party on Tuberculosis of the Spine. *International Orthopaedics* 1999;23:73-81.
108 Mukadi Y, Perriens J, Willame JC, et al. Short Course Antituberculous Therapy for Pulmonary Tuberculosis in Hiv Seropositive Patients A Prospective Controlled Study. *Int Conf AIDS* 1990;507.
109 Narayanan PR. Low rate of emergence of drug resistance in sputum positive patients treated with short course chemotherapy. *Int J Tuberc Lung Dis* 2001;5:40-5.
110 National Cooperative Group On Clinical Study Of Rafapentine. A controlled clinical trial of rifapentine containing chemotherapy regimens for bacillary tuberculosis: Results at three years. *Chinese Journal of Antibiotics* 1992;17:313-22.
111 Nitti V, Delli Veneri F, Marra A. Rifomycin in treatment of chronic tuberculosis with bacilli resistant to standard medications. *Acta Tuberc Pneumol Belg* 1969;60:455-65.
112 Nunn AJ, Cook SV, Burgos M, et al. Results at 30 months of a randomised trial of FDCs and separate drugs for the treatment of tuberculosis. *Int J Tuberc Lung Dis* 2014;18:01.
113 Pamra SP, Nagpaul DR, Baily GVJ, et al. Short course chemotherapy trial involving the use of INH and Thiacetazone in the continuation phase. *Indian J Tuberc* 1988;35:68-75.
114 Perriens JH, Stlouis ME, Mukadi YB, et al. Pulmonary Tuberculosis in Hiv-Infected Patients in Zaire - A Controlled Trial of Treatment for Either 6 Or 12 Months. *N Engl J Med* 1995;332:779-84.
115 Pilheu JA. Short-duration treatment of pulmonary tuberculosis. 1977. *Chest* 2009;136:e30.
116 Poh SC. Controlled Trial of 4 Month and 6 Month Regimens of Chemo Therapy in the Treatment of Pulmonary Tuberculosis A Singapore British Medical Research Council Study. *Bull Int Union Tuberc* 1978;53:242-3.
117 Pult N, Barandun S. Report of the team of the Society of Swiss Tuberculosis Experts on the clinical trial of cycloserine. *Schweiz Z Tuberc Pneumonol* 1958;15:417-68.
118 Punnotok J, Pumprueg U, Chakorn T. A comparison of two short course tuberculosis chemotherapy regimens, both using Rifater during an intensive phase, with a 3 year follow-up. *J Med Assoc Thai* 1995;78:298-304.

119 Rabier M, Sarrat H. Comparison of 3 Ambulatory Methods of Treating Pulmonary Tuberculosis in Senegal Preliminary Results with Thiacetazone. *Rev Tuberc Pneumol (Paris)* 1971;35:751-62.
120 Radenbach KL. Results of Clinical Studies with Capreomycin Ethambutol and Rifampicin in the Heckeshorn Hospital Berlin. *Scand J Respir Dis Suppl* 1969;69:43-53.
121 Ramasamy R, Reginald A, Ganesan E. The use of high-dose isomazid in intermittent regime TB treatment--some preliminary findings. *Trop Doct* 2000;30:56.
122 Research Committee of the British Tuberculosis Association. An investigation of the value of ethionamide with pyrazinamide or cycloserine in the treatment of chronic pulmonary tuberculosis. A report from the Research Committee of the British Tuberculosis Association. *Tubercle* 1961;42:269-86.
123 Research Committee of the Tuberculosis Association of India. Short course chemotherapy of pulmonary tuberculosis - Interim report on second tuberculosis association of India trial. *Indian J Tuberc* 1983;30:57-62.
124 Reves R, Heilig CM, Tapy JM, et al. Intermittent tuberculosis treatment for patients with isoniazid intolerance or drug resistance. *Int J Tuberc Lung Dis* 2014;18:571-80.
125 Rey DR, Guerra Sanz FJ. Clinical trial of short-course chemotherapy regimens (6 months) in patients with newly detected pulmonary tuberculosis. *Bull Int Union Tuberc* 1979;54:30-1.
126 Rist N. The attack treatment of pulmonary tuberculosis with combined isoniazid and ethionamide. The conclusions drawn from the Tangier trial. *Rev Tuberc Pneumol (Paris)* 1962;26:740-5.
127 Rist N. A controlled trial of three regimens of self-administered and supervised chemotherapy for pulmonary tuberculosis. B. Bacteriological report. *Bull Int Union Tuberc* 1970;44:38-53.
128 Robitzek EH. A controlled trial of six months chemotherapy in pulmonary tuberculosis. *Am Rev Respir Dis* 1983;127:255.
129 Rogowski J, Masztalerz J, Piasecki Z, et al. Once Weekly Treatment of Fast Isoniazid Inactivators with Newly Diagnosed Pulmonary Tuberculosis with Drug Combinations Containing Isoniazid Matrix Icn Canada Controlled Clinical Trial. *Bull Int Union Tuberc* 1982;57:21.
130 Ruslami R, Nijland HMJ, Alisjahbana B, et al. Pharmacokinetics and tolerability of a higher rifampin dose versus the standard dose in pulmonary tuberculosis patients. *Antimicrob Agents Chemother* 2007;51:2546-51.
131 Salinger PL, Dormer BA. Rifampicin, ethambutol, ethionamide and hydronsan in advanced pulmonary tuberculosis. *S Afr Med J* 1972;46:354-8.
132 Schutz I. A comparison of rifampicin, ethambutol and PAS in short-term monotherapy. Preliminary results of the third cooperative study of the WATL. *Acta Tuberc Pneumol Belg* 1969;60:437-41.
133 Schutz I, Bartmann K. Sputum excretion of tubercle bacilli as early criterion in the evaluation of the therapeutic effectiveness of antituberculous drugs, illustrated by its use in a controlled trial of single-drug regimens. *Antibiot Chemother* 1970;16:490-500.
134 Shennan DH. A controlled clinical trial of streptomycin plus thiacetazone: British Medica Research Council-Rhodesian Tuberculosis Service-Brompton Hospital laboratory investigation. *Tubercle* 1968;49:8.
135 Shishito S, Sugawara H, Kato M, et al. A clinical trial of rifampicin in renal tuberculosis. *Kekkaku* 1973;48:323-8.
136 Snider DE. A Controlled Trial of 6 Months Chemotherapy in Pulmonary Tuberculosis. *Am Rev Respir Dis* 1983;127:254-5.
137 Snider DE, Graczyk J, Bek E, et al. Supervised six-months treatment of newly diagnosed pulmonary tuberculosis using isoniazid, rifampin, and pyrazinamide with and without streptomycin. *Am Rev Respir Dis* 1984;130:1091-4.

138 Sokolova GB, Khaimenova TI, Borisov SE, et al. The effectiveness and safety of sparfloxacine in combined therapy for tuberculosis. *Probl Tuberk Bolezn Legk* 2007;40-5.
139 Sokolova GB, Kirtaeva OV, Lazareva IV, et al. Russian cycloserine in complex therapy of drug resistant tuberculosis. *Antibiot Khimioter* 2008;53:32-4.
140 Sokolova GB, Koriakin VA, Khalbaeva IV, et al. Combined chemotherapy of patients with tuberculosis - new regimens and dosage forms. *Probl Tuberk* 1993;5:21-3.
141 Somner AR. Short-Course Chemotherapy in Pulmonary Tuberculosis - 3Rd Report. *Lancet* 1980;1:1182-4.
142 Somner AR, ANGEL JH, Citron KM. A controlled trial of six months' chemotherapy in pulmonary tuberculosis - second report: Results up to 24 months following the end of chemotherapy. *Thorax* 1982;37:791-2.
143 Stahle I. Benzacyl and pas in the treatment of pulmonary tuberculosis. A comparative study. *Acta Tuberc Scand* 1957;34:83-101.
144 Steininger WJ. Ethambytol in the retreatment of pulmonary tuberculosis. United States Public Health Service cooperative trial. *Bull Int Union Tuberc* 1968;41:181-2.
145 Sung CC, Chi HS, Young PH. Comparison of 9-month and 6-month short course regimens for sputum microscopy positive pulmonary tuberculosis patients under programme conditions. *Tuberc Respir Dis (Seoul)* 1986;33:9-18.
146 Sutherland AM. The treatment of tuberculosis of the endometrlum. A comparison of streptomycin and PAS with streptomycin and isoniazid. *Journal of obstetrics and gynaecology of the British Empire* 1958;65:1958-450.
147 Swai OB. 3rd study by the African and British Medical Research Councils of short term chemotherapy (including relapse rates during 18 months following chemotherapy). *Bull Int Union Tuberc* 1978;53:254-5.
148 Tala E, Heinivaara O, Kokkola K, et al. 9 Month Chemo Therapy of Isoniazid Plus Rifampicin in Pulmonary Tuberculosis Compared with Initial Triple Regimens A Controlled Cooperative Trial in Finland. *Bull Int Union Tuberc* 1982;57:64.
149 The British Thoracic Society Research Committee. Short course chemotherapy for lymph node tuberculosis: final report at 5 years. British Thoracic Society Research Committee. *British Journal of Diseases of the Chest* 1988;82:282-4.
150 Thwaites GE, Bhavnani SM, Chau TTH, et al. Randomized pharmacokinetic and pharmacodynamic comparison of fluoroquinolones for tuberculous meningitis. *Antimicrob Agents Chemother* 2011;55:3244-53.
151 Tripathy SP. Controlled clinical trial of a 3-month regimen and 2 5-month regimens in the treatment of pulmonary tuberculosis. 2d study of the short-term treatment administered in Madras. *Bull Int Union Tuberc* 1983;58:97-101.
152 Tripathy SP, Menon NK, Mitchison DA, et al. Response to treatment with isoniazid plus PAS of tuberculous patients with primary isoniazid resistance. *Tubercle* 1969;50:257-68.
153 Tuberculosis Association of India. Short-term chemotherapy of pulmonary tuberculosis - A controlled trial. *Indian J Tuberc* 1980;27:48-53.
154 Tuberculosis Chemotherapy Centre Madras. A controlled comparison of a twice-weekly and three once-weekly regimens in the initial treatment of pulmonary tuberculosis. *Bull World Health Organ* 1970;43:143-206.
155 United States Public Health Service Tuberculosis Therapy Trial. Sequential use of paired combinations of isoniazid, streptomycin, paraaminosalicylic acid, and pyrazinamide. A United States Public Health Service Tuberculosis Therapy Trial. *American Review of Respiratory Disease* 1959;80:627-40.
156 Valenzuela P, Valenzuela MT, Ponce J. Response to short-course chemotherapy of patients with initial resistance to antituberculosis drugs. *Bull Pan Am Health Organ* 1988;22:175-83.
157 Velayutham BV, Allaudeen IS, Sivaramakrishnan GN, et al. Sputum culture conversion with moxifloxacin-containing regimens in the treatment of patients with

newly diagnosed sputum-positive pulmonary tuberculosis in South India. *Clin Infect Dis* 2014;59:15.
158 Viswanat R, Singh MM, Sarkar DK, et al. Double-Blind Clinical Trial with Rimactane in Salvage Cases of Pulmonary Tuberculosis. *Indian J Med Res* 1972;60:312.
159 Viswanathan R, Dingley HB, Singh MM. Short-Course Chemotherapy of Pulmonary Tuberculosis 2Nd Trial of the Tuberculosis Association. *Indian Journal of Tuberculosis* 1984;31:81-8.
160 Viswanathan R, Gupta NP, Rao PU, et al. A trial on cycloserine in salvage cases of pulmonary tuberculosis. *Tubercle* 1963;44:351-4.
161 Wang JY, Wang JT, Tsai TH, et al. Adding moxifloxacin is associated with a shorter time to culture conversion in pulmonary tuberculosis. *Int J Tuberc Lung Dis* 2010;14:65-71.
162 Wissenschaftliche Arbeitsgemeinschaft für die Therapie von Lungenkrankheiten. A comparison of rifampicin, ethambutol or PAS in short-term monotherapy in pretreated cavitary pulmonary tuberculosis. *Pneumonologie* 1975;153:43-59.
163 Yamamoto K. Controlled comparison of daily and intermittent treatment regimens with rifampicin. *Bull Int Union Tuberc* 1974;49:442-6.
164 Yan B, Zhu L. Two controlled clinical trials on DL-473-containing regimens in treatment of smear-positive tuberculosis patients. *Tuber Lung Dis* 1994;75:43.
165 Yan BY, Zhu LZ, Chang FY. A Controlled Clinical Trial of Dl-473-Containing Regimens of Chemotherapy for Bacillary Pulmonary Tuberculosis Results at 3 Years. *Am Rev Respir Dis* 1990;141:A435.
166 Yan B-Y, Zhu L-Z, Na X-K, et al. Comparative clinical trial studies on cyclopentyl rifamycin in treatment for pulmonary tuberculosis. *Zhongguo Kang Sheng Su Za Zhi* 1987;12:321-8.
167 Yang SP, Luh KT, Wu MH. A clinical trial of ethambutol in the retreatment of pulmonary tuberculosis--mostly on ambulatory basis. *Taiwan yi xue hui za zhi* 1968;67:348-58.
168 Young PH, Sung CC, Sung CK. Comparison of initial treatment regimen including short course chemotherapy for pulmonary tuberculosis under programme conditions. [Korean]. *Tuberculosis and Respiratory Diseases*29:1982.
169 Youssef HH, Mahmoud E, El MA, et al. Evaluation of the results of rifampicin ethambutol trial in chronic pulmonary tuberculosis. *Ain Shams Med J* 1974;25:124-32.
170 Yu D, Wang J, Hu X. Clinical research of pasinizid on retreated sputum positive pulmonary tuberculosis in senilities. *Zhonghua Jie He He Hu Xi Za Zhi* 2001;24:608-10.
171 Zhang L-X, Kan G-Q, Tu D-H, et al. Fixed-dose combination chemotherapy versus multiple, single-drug chemotherapy for tuberculosis. *Curr Ther Res Clin Exp* 1996;57:849-56.
