# Supplementary material for: What are the most efficacious treatment regimens for isoniazid-resistant tuberculosis? A systematic review and network meta-analysis
Source: Thorax. 2016 Jun 13;71(10):940–9. doi: 10.1136/thoraxjnl-2015-208262 (PMC5036252; doi:10.1136/thoraxjnl-2015-208262)
Supplement: Supplementary file 7 [file thoraxjnl-2015-208262supp_file7.pdf]

SUPPLEMENTARY FILE 7: Quality assessment

Quality assessment of the studies in Supplementary file 5 risk of bias from the six domains using the framework of Higgins *et al.*[1]. *Jindani [2] updates data from both of these studies. AWG/BMRC- Algerian Working Group/British Medical Research Council Cooperative Study, BMRC/HKCS- British Medical Research Council/Hong Kong Chest Service, BMRC/STS- British Medical Research Council Singapore Tuberculosis Service, EA/BMRC- East African/British Medical Research Council, EAMRC- East African Medical Research Council, ECA/BMRC- East and Central African/British Medical Research Council, HKCS- Hong Kong Chest Service, HKCS/BMRC- Hong Kong Chest Service/British Medical Research Council, HKCS/MBMRC- Hong Kong Chest Service/Madras/British Medical Research Council, HKCS/TRC/BMRC- Hong Kong Chest Service/Tuberculosis Research Centre Madras/British Medical Research Council, HKTTS/BMRC- Hong Kong Tuberculosis Treatment Services/British Medical Research Council, ITRC- Indian Tuberculosis Research Centre, KZBMRC- Kenyan Zambian British Medical Research Council, NCGCSR- National Cooperative Group on Clinical Study Of Rifapentine, NRITP- National Research Institute for Tuberculosis Poland, RCBTA- Research Committee of the British Tuberculosis Association, STS- Singapore Tuberculosis Service, STS/BH/BMRC- Singapore Tuberculosis Services/Brompton Hospital/British Medical Research Council, STS/BMRC- Singapore Tuberculosis Service/British Medical Research Council, TBMRC- Tanzania/British Medical Research Council, TCC- Tuberculosis Chemotherapy Centre Madras, TRC- Tuberculosis Research Centre Madras, WATL- Wissenschaftliche Arbeitsgemeinschaft für die Therapie von Lungenkrankheiten, WHOCCTC- World Health Organization Collaborating Centre for Tuberculosis Chemotherapy Prague

Author	Random sequence generation	Allocation concealment	Blinding of participants and personnel	Blinding of outcome assessment	Attrition	Selective reporting
Agounitestane[3]<br>Bellabas[4]<br>Chaulet[5]	Unknown	Unknown	Unknown	Unknown	High	High
Allan[6]<br>HKCS[7]<br>HKTTS/BMRC[8]	Unknown	Unknown	Unknown	Unknown	High	High
Allan[9]<br>HKCS[10]<br>HKCS/BMRC[11]	Unknown	Unknown	Unknown	Unknown	High	High
Andrews[12]<br>Devadatta[13]<br>TCC[14]<br>TCC[15]<br>Velu[16]	Low	Low	Unknown	Unknown	Low	High
Aquinas[17]<br>BMRC/HKCS[18]<br>HKCS[19]<br>Pang[20]	Unknown	Unknown	Unknown	Unknown	High	High
AWG/BMRC[21]	Unknown	Unknown	Unknown	Unknown	High	High
Babu Swai[22]	Unknown	High	Unknown	Unknown	High	Low
Balasubramanian [23]<br>Prabhakar[24]	Unknown	Unknown	Unknown	Unknown	Low	High

Author	Random sequence generation	Allocation concealment	Blinding of participants and personnel	Blinding of outcome assessment	Attrition	Selective reporting
Berkani[25]<br>Berkani[26]	Unknown	Low	Unknown	Unknown	Low	High
Bignall[27]	Unknown	Unknown	Unknown	Unknown	High	High
BMRC/STS[28]<br>Girling[29]	Unknown	Unknown	Unknown	Unknown	Low	High
Boszormenyi[30]	Unknown	Unknown	Unknown	Unknown	Unknown	Low
Boulahbal[31]<br>Chaulet[32]<br>Mazouni[33]	Unknown	Unknown	Unknown	Unknown	High	High
Chukanov[34]	Unknown	Low	Unknown	Unknown	Low	High
Citron[35]<br>RCBTA[36]	Low	Unknown	Unknown	Unknown	High	Low
Donomae[37]	Unknown	Low	Unknown	Unknown	Low	Low
EA/BMRC[38]	Low	Low	Unknown	Unknown	High	High
EA/BMRC[39]<br>EA/BMRC[40]	Low	Low	Unknown	Unknown	Low	High
EA/BMRC[41]	Unknown	Unknown	Unknown	Unknown	High	Low
EA/BMRC[42]<br>EA/BMRC[43]<br>EA/BMRC[44]	Unknown	Low	Unknown	Unknown	High	High
EA/BMRC[45]<br>EA/BMRC[46]<br>Girling[47]	Unknown	Unknown	Unknown	Unknown	High	High
EABMRC*[48]<br>EABMRC[49]<br>EAMRC[50]<br>Jindani[51]<br>Karuga[52]	Unknown	Low	Unknown	Unknown	High	High
EABMRC*[53]<br>EABMRC[54]	Unknown	Low	Unknown	Unknown	High	High
EABMRC[55]<br>EAMRC[56]	Unknown	Low	Unknown	Unknown	High	High
ECA/BMRC[57]<br>ECA/BMRC[58]	Unknown	Low	Unknown	Unknown	High	High

Author	Random sequence generation	Allocation concealment	Blinding of participants and personnel	Blinding of outcome assessment	Attrition	Selective reporting
Fox[59]<br>Girling[60]<br>STS/BMRC[61]<br>Tan[62]	Unknown	Low	Unknown	Unknown	High	High
Fraga[63]	Unknown	Unknown	Unknown	Unknown	Low	High
Girling[64]<br>Horsfall[65]	Unknown	Unknown	Unknown	Unknown	High	Low
Girling[66]	Unknown	Unknown	Unknown	Unknown	High	High
Hetrick[67]	Unknown	Unknown	Unknown	Unknown	High	Low
HKCS[68]<br>HKCS/BMRC[69]<br>HKCS/TRC/BMRC[70]<br>TRC/BMRC[71]	Unknown	Unknown	Unknown	Unknown	High	High
HKCS[72]	Unknown	Unknown	Unknown	Unknown	High	High
Hong[73]	Unknown	Unknown	Unknown	Unknown	High	High
ITRC[74]<br>Santha[75]<br>Tripathy[76]	Unknown	Unknown	Unknown	Unknown	Low	High
Jindani[77]<br>Nunn[78]	Low	Low	High	High	High	High
KZBMRC[79]	Unknown	Low	Low	Low	High	High
Larbaoui[80]	Unknown	Unknown	Unknown	Unknown	Low	Low
Mathew[81]	Unknown	Unknown	Unknown	Unknown	Low	High
Nazareth[82]<br>Ramakrishnan[83]<br>TCC[84]<br>TCC[85]	Low	Low	Unknown	Unknown	Low	High
NCGCSR[86]	Unknown	Unknown	Unknown	Unknown	Low	High
NRITP[87]<br>Zierski[88]<br>Zierski[89]<br>Zierski[90]	Unknown	Unknown	Unknown	Unknown	High	Low
Park[91]	Low	Unknown	High	Unknown	Low	High
Santha[92]<br>TRC[93]	Unknown	Low	Unknown	Unknown	High	High
Sriyabhaya[94]	Unknown	Unknown	Unknown	Unknown	High	Low

**Author**	**Random sequence generation**	**Allocation concealment**	**Blinding of participants and personnel**	**Blinding of outcome assessment**	**Attrition**	**Selective reporting**
STS[95]<br>STS/BH/ BMRC[96]	Unknown	Unknown	Low (for two arms)	Unknown	High	High
STS[97]<br>STS[98]<br>STS[99]<br>Teo[100]	Unknown	Low	Unknown	Unknown	Low	High
STS[101]<br>Teo[102]	Unknown	Unknown	Unknown	Unknown	High	High
Sundberg[103]	Unknown	Low	Unknown	Unknown	High	High
Swaminathan[104]	Low	Low	High	High	Low	High
Tam[105]<br>Tam[106]<br>Tam[107]	Unknown	Low	High	High	Low	High
TBMRC[108]	Unknown	Low	Unknown	Unknown	High	High
TBMRC[109]	Unknown	Low	Unknown	Unknown	High	High
TCC[110]	Unknown	Unknown	Unknown	Unknown	Low	High
Tripathy[111]<br>Tripathy[112]	Unknown	Unknown	Unknown	Unknown	High	High
Velu[113]	Low	Low	Unknown	Unknown	High	Low
WATL[114]	Unknown	Low	Unknown	Unknown	High	Low
WHOCCTC[115]<br>WHOCCTC[116]	Unknown	Unknown	Unknown	Unknown	High	High
Young[117]	Unknown	Unknown	High	High	High	Low
Zierski[118]<br>Zierski[119]	Unknown	Unknown	Unknown	Unknown	High	High

References

1. Higgins JP, Altman DG, Gotzsche PC, et al. The Cochrane Collaboration's tool for assessing risk of bias in randomised trials. *Br Med J* 2011;343:d5928.
2. Jindani A. Short-course (6-month) treatment of pulmonary tuberculosis (Second East African/British Medical Research Council Study). *Bull Int Union Tuberc* 1976;51:53-6.
3. Agounitestane D, Chiheb M, Khaled S, et al. A Therapeutic Trial of A Combination of 3 Essential Drugs in the Short Course of Chemotherapy for Tuberculosis - Results 6 Months After the End of Treatment. *Rev Mal Respir* 1990;7:209-13.
4. Bellabas M, Khaled S, Khaled NA, et al. A Therapeutic Trial of the Combination of Isoniazid, Rifampicin and Pyrazinamide in the 1St 2 Months of Treatment for Pulmonary Tuberculosis. *Revue des Maladies Respiratoires* 1989;6:59-64.
5. Chaulet P, Boulahbal F. Clinical trial of a combination of three drugs in fixed proportions in the treatment of tuberculosis. Groupe de Travail sur la Chimiotherapie de la Tuberculose. *Tuber Lung Dis* 1995;76:407-12.
6. Allan WGL, Chan YL, Singh D. Controlled trial of 6- and 9-month regimens of daily and intermittent streptomycin plus isoniazid plus pyrazinamide for pulmonary tuberculosis in Hong Kong. *Tubercle* 1975;56:81-96.
7. Hong Kong Chest Service., British Medical Research Council. Controlled Trial of 6 Month and 9 Month Regimens of Daily and Intermittent Streptomycin Plus Isoniazid Plus Pyrazinamide for Pulmonary Tuberculosis in Hong-Kong the Results Up to 30 Months. *American Review of Respiratory Disease* 1977;115:727-35.
8. Hong Kong Tuberculosis Treatment Services/British Medical Research Council. Adverse reactions to short-course regimens containing streptomycin, isoniazid, pyrazinamide and rifampicin in Hong Kong. *Tubercle* 1976;57:81-95.
9. Allan WGL. Controlled Trial of 4 3-Times-Weekly Regimens and A Daily Regimen All Given for 6-Months for Pulmonary Tuberculosis .2. the Results Up to 24-Months. *Tubercle* 1982;63:89-98.
10. Hong Kong Chest Service., British Medical Research Council. Controlled Trial of 4 Thrice Weekly Regimens and A Daily Regimen All Given for 6 Months for Pulmonary Tuberculosis. *Lancet* 1981;1:171-4.
11. Hong Kong Chest Service/British Medical Research Council. Five-year follow-up of a controlled trial of five 6-month regimens of chemotherapy for pulmonary tuberculosis. Hong Kong Chest Service/British Medical Research Council. *The American review of respiratory disease* 1987;136:1339-42.
12. ANDREWS RH, Devadatta S, Fox W, et al. Prevalence of tuberculosis among close family contacts of tuberculous patients in South India, and influence of segregation of the patient on early attack rate. *Bull World Health Organ* 1960;23:463-510.
13. Devadatta S, Bhatia AL, ANDREWS RH, et al. Response of patients infected with isoniazid-resistant tubercle baccilli to treatment with isoniazid plus PAS or isoniazid alone. *Bull World Health Organ* 1961;25:807-29.
14. Tuberculosis Chemotherapy Centre M. A concurrent comparison of isoniazid plus PAS with three regimens of isoniazid alone in the domiciliary treatment of pulmonary tuberculosis in South India. *Bull World Health Organ* 1960;23:535-85.
15. Tuberculosis Chemotherapy Centre M. A concurrent comparison of home and sanatorium treatment of pulmonary tuberculosis in South India. *Bull World Health Organ* 1959;21:51-144.
16. Velu S, ANDREWS RH, ANGEL JH, et al. Progress in the second year of patients with quiescent pulmonary tuberculosis after a year of domiciliary chemotherapy, and influence of further chemotherapy on the relapse rate. *Bull World Health Organ* 1960;23:511-33.
17. Aquinas SM. Controlled Trial of 6 Month and 8 Month Regimens of Chemo Therapy in the Treatment of Pulmonary Tuberculosis A Hong-Kong Chest Service British Medical Research Council Study. *Bull Int Union Tuberc* 1978;53:237-9.

18 British Medical Research Council-Hong Kong Chest Service. Controlled Trial of 6 Month and 8 Month Regimens in the Treatment of Pulmonary Tuberculosis 1st Report. *Am Rev Respir Dis* 1978;118:219-28.
19 Hong Kong Chest Service, British Medical Research Council. Controlled Trial of 6 Month and 8 Month Regimens in the Treatment of Pulmonary Tuberculosis the Results Up to 24 Months. *Tubercle* 1979;60:201-10.
20 Pang SC. Preliminary-Results of Hong-Kong-British-Medical-Research-Council 2Nd Controlled Trial of Short-Course Anti-Tuberculosis Regimens. *Tubercle* 1978;59:64.
21 Algerian Working Group/British Medical Research Council Cooperative Study. Short-course chemotherapy for pulmonary tuberculosis under routine programme conditions: a comparison of regimens of 28 and 36 weeks duration in Algeria. *Tubercle* 1991;72:88-100.
22 Babu Swai O, Aluoch JA, Githui WA, et al. Controlled Clinical-Trial of A Regimen of 2 Durations for the Treatment of Isoniazid Resistant Pulmonary Tuberculosis. *Tubercle* 1988;69:5-14.
23 Balasubramanian R, Sivasubramanian S, Vijayan VK, et al. Five year results of a 3-month and two 5-month regimens for the treatment of sputum-positive pulmonary tuberculosis in south India. *Tubercle* 1990;71:253-8.
24 Prabhakar R. A Controlled Clinical-Trial of 3-Month and 5-Month Regimens in the Treatment of Sputum-Positive Pulmonary Tuberculosis in South-India. *Am Rev Respir Dis* 1986;134:27-33.
25 Berkani M, Chaulet P, Darbyshire JH. Controlled clinical trial comparing a 6-month and a 12-month regimen in the treatment of pulmonary tuberculosis in the Algerian Sahara. *Am Rev Respir Dis* 1984;129:921-8.
26 Berkani M, Chaulet P, Darbyshire JH, et al. Results of a therapeutic trial comparing a 6-month regimen to a 12-month regimen in the treatment of pulmonary tuberculosis in the Algerian Sahara. Final report: results 3 years after the onset of treatment. *Rev Mal Respir* 1986;3:73-85.
27 Bignall JR. The Effect of Adding Thiacetazone Or P Amino Salicylic-Acid to the Regimen of Twice Weekly Streptomycin and Isoniazid in Patients with Pulmonary Tuberculosis An International Controlled Trial. *Bull Int Union Tuberc* 1974;49:7-24.
28 British Medical Research Council-Singapore Tuberculosis Service. Controlled Trial of Intermittent Regimens of Rifampicin Plus Isoniazid for Pulmonary Tuberculosis in Singapore. *Lancet* 1975;2:1105-9.
29 Girling DJ, Nunn AJ, Fox W, et al. Controlled Trial of Intermittent Regimens of Rifampin Plus Isoniazid for Pulmonary Tuberculosis in Singapore - Results Up to 30 Months. *Am Rev Respir Dis* 1977;116:807-20.
30 Boszormenyi M. Controlled clinical trials with Isoxyl. *Antibiot Chemother* 1970;16:124-7.
31 Boulahbal F, Tazir M, Khaled S, et al. Short Duration Chemotherapy of Pulmonary Tuberculosis Bacteriological Results. *Arch Inst Pasteur Alger* 1980;54:162-72.
32 Chaulet P, Boulahbal F, Khaled NA, et al. A Comparison of 3 6 Month Regimes for Pulmonary Tuberculosis in Routine Clinic Practice in Algiers - 18 Month Results. *Rev Fr Mal Respir* 1983;11:667-76.
33 Mazouni L, Tazir M, Boulahbal F, et al. Controlled study comparing 3 daily chemotherapy regimens for six months in pulmonary tuberculosis in routine practice in Algiers. Results at 30 months. *Rev Mal Respir* 1985;2:209-14.
34 Chukanov VI, Komissarova OG, Maishin VI, et al. Efficiency of a new standard chemotherapy regimen in the treatment of patients with recurrent pulmonary tuberculosis. *Probl Tuberk Bolezn Legk* 2006;9-13.
35 Citron KM. Therapy of Tuberculosis - British Tuberculosis Association Trial of Ethionamide, Pyrazinamide, and Cycloserine in Treatment of Drug-Resistant Pulmonary Tuberculosis. *Am Rev Respir Dis* 1963;88:113.

36 Research Committee of the British Tuberculosis Association. Ethionamide, Pyrazinamide and Cycloserine in the Treatment of Drug-Resistant Pulmonary Tuberculosis. *Tubercle* 1963;44:195-214.
37 Donomae I, Yamamot K. Clinical Evaluation of Ethambutol in Pulmonary Tuberculosis. *Ann N Y Acad Sci* 1966;135:849.
38 East African/British Medical Research Council. Comparative trial of isoniazid in combination with thiacetazone or a substituted diphenylthiourea (su 1906) or pas in the treatment of acute pulmonary tuberculosis in east africans. A cooperative investigation in east African hospitals and laboratories with the collaboration of the british medical research council. *Tubercle* 1960;41:1960.
39 East African British Medical Research Council. INFLUENCE OF PRETREATMENT BACTERIAL RESISTANCE TO ISONIAZID, THIACETAZONE OR PAS ON THE RESPONSE TO CHEMOTHERAPY OF AFRICAN PATIENTS WITH PULMONARY TUBERCULOSIS. *Tubercle* 1963;44:393-416.
40 East African and British Medical Research Councils. Isoniazid with Thiacetazone in the Treatment of Pulmonary Tuberculosis in East Africa- Second Investigation. *Tubercle* 1963;44:301-32.
41 East African/British Medical Research Council. A comparison of two regimens of streptomycin plus PAS in the retreatment of pulmonary tuberculosis. *Tubercle* 1968;49:70-8.
42 East African/British Medical Research Council. Isoniazid with thiacetazone (thioacetazone) in the treatment of pulmonary tuberculosis in East Africa--fifth investigation. A co-operative study in East African hospitals, clinics and laboratories with the collaboration of the East African and British Medical Research Councils. *Tubercle* 1970;51:123-51.
43 East African/British Medical Research Council. Isoniazid with thiacetazone (thioacetazone) in the treatment of pulmonary tuberculosis in East Africa. Third Report of Fifth Investigation. A co-operative study in East Africian hospitals, clinics and laboratories with the collaboration of the East African and British Medical Research Councils. *Tubercle* 1973;54:169-79.
44 East African/British Medical Research Council. Isoniazid with thiacetazone (thioacetazone) in the treatment of pulmonary tuberculosis in East Africa--second report of fifth investigation. A co-operative study in East African hospitals, clinics and laboratories with the collaboration of the East African and British Medical Research Councils. *Tubercle* 1970;51:353-8.
45 East African/British Medical Research Council. Controlled clinical trial of four short-course regimens of chemotherapy for two durations in the treatment of pulmonary tuberculosis. Second report. Third East African/British Medical Research Council Study. *Tubercle* 1980;61:59-69.
46 Third East African-British Medical Research Council Study. Controlled Clinical Trial of 4 Short-Course Regimens of Chemo Therapy for 2 Durations in the Treatment of Pulmonary Tuberculosis Part 1. *Am Rev Respir Dis* 1978;118:39-48.
47 Girling DJ. Preliminary-Results of East-African-British-Medical-Research-Councils 3Rd Controlled Trial of Short-Course Anti-Tuberculosis Regimens. *Tubercle* 1978;59:65.
48 East African British Medical Research Councils. Controlled clinical trial of four short-course (6-month) regimens of chemotherapy for treatment of pulmonary tuberculosis. Third report. East African-British Medical Research Councils. *Lancet* 1974;2:237-40.
49 East African British Medical Research Council. Controlled Clinical Trial of Short Course 6 Month Regimens of Chemo Therapy for Treatment of Pulmonary Tuberculosis. *Lancet* 1972;1:1079-85.
50 East African Medical Research Council, British Medical Research Council. Controlled Clinical Trial of 4 Short Course 6 Month Regimens of Chemo Therapy for Treatment of Pulmonary Tuberculosis 2Nd Report. *Lancet* 1973;1:1331-8.

51 Jindani A, Heffernan JF. A Controlled Clinical Trial in East Africa of 4 6 Month Regimens and A Standard 18 Month Regimen of Chemo Therapy Results in the 1St 18 Months. *British Thoracic and Tuberculosis Association Review* 1973;3:27.
52 Karuga WK. Short-term treatment of pulmonary tuberculosis. *Bull Int Union Tuberc* 1974;49:388-97.
53 Second East African British Medical Research Council Study. Controlled Clinical Trial of 4 6 Month Regimens of Chemo Therapy for Pulmonary Tuberculosis. *Am Rev Respir Dis* 1976;114:471-5.
54 East African British Medical Research Council. Controlled clinical trial of four short-course (6-month) regimens of chemotherapy for treatment of pulmonary tuberculosis. *Lancet* 1974;2:1100-6.
55 East African and British Medical Research Councils. Controlled clinical trial of five short-course (4-month) chemotherapy regimens in pulmonary tuberculosis. First report of 4th study. *Lancet* 1978;2:334-8.
56 East-African British Medical Research Council. Controlled Clinical Trial of 5 Short Course 4 Month Chemo Therapy Regimens in Pulmonary Tuberculosis 2Nd Report of the 4Th Study. *American Review of Respiratory Disease* 1981;123:165-70.
57 East and Central African/British Medical Research Council. Controlled clinical trial of 4 short-course regimens of chemotherapy (three 6-month and one 8-month) for pulmonary tuberculosis: final report. East and Central African/British Medical Research Council Fifth Collaborative Study. *Tubercle* 1986;67:5-15.
58 East Central African British Medical Research Council. Controlled Clinical Trial of 4 Short Course Regimens of Chemo Therapy Three 6 Month and One 8 Month for Pulmonary Tuberculosis. *Tubercle* 1983;64:153-66.
59 Fox W, Kee TS. Clinical trial of three 6-month regimens of chemotherapy given intermittently in the continuation phase in the treatment of pulmonary tuberculosis. *Am Rev Respir Dis* 1985;132:374-8.
60 Girling DJ. Singapore-Tuberculosis-Service and British-Medical-Research-Council Trial of 3 6-Month Regimens for Pulmonary Tuberculosis Given 3 Times Weekly in the Continuation Phase - Results Up to 30 Months. *Thorax* 1984;39:720.
61 Singapore Tuberculosis Service-British Medical Research Council. Five-Year Follow-Up of A Clinical Trial of Three 6-Month Regimens of Chemotherapy Given Intermittently in the Continuation Phase in the Treatment of Pulmonary Tuberculosis. *Am Rev Respir Dis* 1988;137:1147-50.
62 Tan T. Comparison of three 6-month regimens for smear-positive cases of pulmonary tuberculosis: A Singapore Government Tuberculosis Service/British Medical Research Council Investigation. *Bull Int Union Tuberc* 1984;59:14-5.
63 Fraga H, Gomes O, Paz de Almeida A, et al. Comparative study (in a controlled therapeutic trial) of 3 intermittent regimens, after an initial period of daily administration, in the repeated treatment of pulmonary tuberculosis (results). *Bull Int Union Tuberc* 1973;48:125-38.
64 Girling DJ. Daily and intermittent regimens using ethambutol and rifampicin in the treatment of unresponsive cases. (Evaluation after 1 year.) II. Side effects. *Bull Int Union Tuberc* 1974;49:451-5.
65 Horsfall PA. Daily and intermittent regimens using ethambutol and rifampicin in the treatment of unresponsive cases. (Evaluation after 1 year.) I. Clinical course. *Bull Int Union Tuberc* 1974;49:447-51.
66 Girling DJ, Chan SL. Controlled trial of 2, 4, and 6 months of pyrazinamide in 6-month, three-times-weekly regimens for smear-positive pulmonary tuberculosis, including an assessment of a combined preparation of isoniazid, rifampin, and pyrazinamide: Results at 30 months. *Am Rev Respir Dis* 1991;143:700-6.
67 Hetrick C, Ras R, Turri M. Combined rifampicin-ethambutol therapy of resistent pulmonary tuberculosis. Effectiveness of various rifampicin doses in combination with ethambutol. *Dtsch Med Wochenschr* 1970;95:1830-3.

68 Hong Kong Chest Service. A study of the characteristics and course of sputum smear-negative pulmonary tuberculosis. *Tubercle* 1981;62:155-67.
69 Hong Kong Chest Service, Madras-British Medical Research Council. A Controlled Trial of 2 Month 3 Month and 12 Month Regimens of Chemo Therapy for Sputum Smear Negative Pulmonary Tuberculosis Results at 30 Months. *Am Rev Respir Dis* 1981;124:138-42.
70 Hong Kong Chest Service TRCMIaBMRC. Sputum-smear-negative pulmonary tuberculosis. Controlled trial of 3-month and 2-month regimens of chemotherapy. First report. *Lancet* 1979;1:1979.
71 Tuberculosis Research Centre Madras British Medical Research Council. A Controlled Trial of 2 Month and 12 Month Regimens of Chemo Therapy for Sputum Smear Negative Pulmonary Tuberculosis Results at 60 Months. *American Review of Respiratory Disease* 1984;130:23-8.
72 Hong Kong Chest Service, Tuberculosis Research Centre Madras, British Medical Research Council. A Controlled Trial of 3-Month 4-Month and 6-Month Regimens of Chemotherapy for Sputum-Smear-Negative Pulmonary Tuberculosis Results at 5 Years. *Am Rev Respir Dis* 1989;139:871-6.
73 Hong YP, Kim SC, Chang SC, et al. Comparison of A Daily and 3 Intermittent Retreatment Regimens for Pulmonary Tuberculosis Administered Under Program Conditions. *Tubercle* 1988;69:241-53.
74 Tuberculosis Research Centre I. Study of Chemo Therapy Regimens of 5 and 7 Months Duration and the Role of Cortico Steroids in the Treatment of Sputum Positive Patients with Pulmonary Tuberculosis in South India. *Tubercle* 1983;64:73-92.
75 Santha T, Nazareth O, Krishnamurthy MS, et al. Treatment of pulmonary tuberculosis with short course chemotherapy in South India - 5-year follow up. *Tubercle* 1989;70:229-34.
76 Tripathy SP. Madras Study of Short-Course Chemotherapy in Pulmonary Tuberculosis. *Bull Int Union Tuberc* 1979;54:28-30.
77 Jindani A, Nunn AJ, Enarson DA. Two 8-month regimens of chemotherapy for treatment of newly diagnosed pulmonary tuberculosis: international multicentre randomised trial. *Lancet* 2004;364:1244-51.
78 Nunn AJ, Jindani A, Enarson DA. Results at 30 months of a randomised trial of two 8-month regimens for the treatment of tuberculosis. *Int J Tuberc Lung Dis* 2011;15:741-5.
79 Kenyan Zambian British Medical Research Council. Controlled Clinical Trial of Levamisole in Short-Course Chemotherapy for Pulmonary Tuberculosis A Kenyan Zambian British Medical Research Council Collaborative Study. *Am Rev Respir Dis* 1989;140:990-5.
80 Larbaoui D, Chaulet P, Grosset J, et al. Intermittent treatment of "intractable chronic tuberculosis" by combined rifampicin-ethambutol: preliminary results of a controlled survey made in Algeria. *Rev Tuberc Pneumol (Paris)* 1970;34:559-66.
81 Mathew R, Rehman F, Santha T, et al. A controlled clinical trial of oral short-course regimens in the treatment of sputum-positive pulmonary tuberculosis. *Int J Tuberc Lung Dis* 1997;1:509-17.
82 Nazareth O, Devadatta S, Evans C, et al. A two-year follow-up of patients with quiescent pulmonary tuberculosis following a year of chemotherapy with an intermittent (twice-weekly) regimen of isoniazid plus streptomycin or a daily regimen of isoniazid plus PAS. *Tubercle* 1966;47:178-89.
83 Ramakrishnan C, V, Devadatta S, Evans C, et al. A 4 Year Follow-Up of Patients with Quiescent Pulmonary Tuberculosis at the End of A Year of Chemo Therapy with Twice Weekly Isoniazid Plus Streptomycin Or Daily Isoniazid Plus Para Amino Salicylic-Acid. *Tubercle* 1969;50:115-24.

84 Tuberculosis Chemotherapy Centre M. A concurrent comparison of intermittent (twice-weekly) isoniazid plus streptomycin and daily isoniazid plus PAS in the domiciliary treatment of pulmonary tuberculosis. *Bull Wld Hlth Org* 1964;31:247-71.
85 Tuberculosis Chemotherapy Centre M. Intermittent treatment of pulmonary tuberculosis. A concurrent comparison of twice-weekly isoniazid plus streptomycin and daily isoniazid plus p-aminosalicylic acid in domiciliary treatment. *Lancet* 1963;281:1078-80.
86 National Cooperative Group On Clinical Study Of Rifapentine. Controlled clinical trial of rifapentine given once weekly or fortnightly in 6-month regimes for the treatment of bacillary tuberculosis: Results at 3-year follow-up. *Zhongguo Kang Sheng Su Za Zhi* 1997;22:423-30.
87 National Research Institute for Tuberculosis P. A comparative study of daily followed by twice- or once-weekly regimens of ethambutol and rifampicin in the retreatment of patients with pulmonary tuberculosis: second report. *Tubercle* 1976;57:105-13.
88 Zierski M, Bek E, Bergson H, et al. Retreatment of chronic pulmonary tuberculosis with regimens including high and low doses of rifampicin in the intermittent phase recent and late results - a controlled comparison study. *Bull Int Union Tuberc* 1976;51:121-6.
89 Zierski M. A trial of intermittent rifampicin and ethambutol in retreatment regimens. *Scand J Respir Dis* 1973;54:132-5.
90 Zierski M, Bek Z, Kucharska A. A comparative study of daily followed by twice or once weekly regimens of ethambutol and rifampicin in retreatment of patients with pulmonary tuberculosis. The results at 1 year. *Tubercle* 1975;56:1-26.
91 Park SH, Yang SK, Yang DH, et al. Prospective Randomized Trial of Six-Month versus Nine-Month Therapy for Intestinal Tuberculosis. *Antimicrob Agents Chemother* 2009;53:4167-71.
92 Santha T, Rehman F, Mitchison DA, et al. Split-drug regimens for the treatment of patients with sputum smear-positive pulmonary tuberculosis - a unique approach. *Trop Med Int Health* 2004;9:551-8.
93 Tuberculosis Research Centre M. Interim findings on the evaluation of split drug regimens for pulmonary tuberculosis - A randomised controlled clinical trial. *Indian Journal of Tuberculosis* 1995;42:201-6.
94 Sriyabhaya N, Jittinandana A, Kecharanantana P. Ambulatory intermittent rifampicin and ethambutol in the retreatment of pulmonary tuberculosis. *J Med Assoc Thai* 1974;57:551-7.
95 Singapore Tuberculosis Services. A Controlled Clinical Trial of the Role of Thiacetazone Containing Regimens in the Treatment of Pulmonary Tuberculosis in Singapore. *Tubercle* 1971;52:88-116.
96 Singapore Tuberculosis Services/Brompton Hospital/British Medical Research Council. A controlled clinical trial of the role of thiacetazone containing regimens in the treatment of pulmonary tuberculosis in Singapore: second report. *Tubercle* 1974;55:251-60.
97 Singapore Tuberculosis Service British Medical Research Council. Long-Term Follow-Up of A Clinical Trial of Six-Month and Four-Month Regimens of Chemotherapy in the Treatment of Pulmonary Tuberculosis. *Am Rev Respir Dis* 1986;133:779-83.
98 Singapore Tuberculosis Service, British Medical Research Council. Clinical Trial of 6 Month and 4 Month Regimens of Chemo Therapy in the Treatment of Pulmonary Tuberculosis Results Up to 30 Months. *Tubercle* 1981;62:95-102.
99 Singapore Tuberculosis Service, British Medical Research Council. Clinical Trial of 6 Month and 4 Month Regimens of Chemo Therapy in the Treatment of Pulmonary Tuberculosis. *Am Rev Respir Dis* 1979;119:579-86.
100 Teo SK, Tan TH. Long-Term Follow Up of A Clinical-Trial of 6 Month and 4 Month Regimens of Chemotherapy in the Treatment of Pulmonary Tuberculosis. *Thorax* 1985;40:696.

101 Singapore Tuberculosis Service, British Medical Research Council. Assessment of A Daily Combined Preparation of Isoniazid Rifampin and Pyrazinamide in A Controlled Trial of Three 6-Month Regimens for Smear-Positive Pulmonary Tuberculosis. *Am Rev Respir Dis* 1991;143:707-12.
102 Teo SK. Assessment of a combined preparation of isoniazid, rifampicin and pyrazinamide (Rifater (R)) in the initial phase of chemotherapy in three 6-month regimens for smear-positive pulmonary tuberculosis: a five-year follow-up report. *Int J Tuberc Lung Dis* 1999;3:126-32.
103 Sundberg T. A controlled trial of ethionamide with isoniazid in the treatment of pulmonary tuberculosis in hong kong. *Tubercle* 1964;45:299-320.
104 Swaminathan S, Narendran G, Venkatesan P, et al. Efficacy of a 6-month versus 9-month intermittent treatment regimen in HIV-infected patients with tuberculosis: A randomized clinical trial. *Am J Respir Crit Care Med* 2010;181:01.
105 Tam CM, Chan SL, Kam KM, et al. Rifapentine and isoniazid in the continuation phase of a 6-month regimen. Final report at 5 years: Prognostic value of various measures. *Int J Tuberc Lung Dis* 2002;6:3-10.
106 Tam CM, Chan SL, Kam KM, et al. Rifapentine and isoniazid in the continuation phase of a 6-month regimen. Interim report: No activity of isoniazid in the continuation phase. *Int J Tuberc Lung Dis* 2000;4:262-7.
107 Tam CM, Chan SL, Lam CW, et al. Rifapentine and isoniazid in the continuation phase of treating pulmonary tuberculosis - Initial report. *Am J Respir Crit Care Med* 1998;157:1726-33.
108 Tanzania/British Medical Research Council. A controlled trial of a 4-weekly supplement of rifampicin, pyrazinamide and streptomycin in the continuation phase of a 7-month daily chemotherapy regimen for pulmonary tuberculosis. *S Afr Med J* 1996;86:960-5.
109 Tanzania-British Medical Research Council Study. Controlled Clinical Trial of Two 6-Month Regimens of Chemotherapy in the Treatment of Pulmonary Tuberculosis. *Am Rev Respir Dis* 1985;131:727-31.
110 Tuberculosis Chemotherapy Centre M. A controlled comparison of two fully supervised once-weekly regimens in the treatment of newly diagnosed pulmonary tuberculosis. *Tubercle* 1973;54:23-45.
111 Tripathy SP. Madras study on intermittent oral chemotherapy using ethambutol and isoniazid. *Bull Int Union Tuberc* 1974;49:427-34.
112 Tripathy SP, Ramakrishnan C, V, Devadatta S, et al. Ethambutol Plus Isoniazid for the Treatment of Pulmonary Tuberculosis A Controlled Trial of 4 Regimens. *Tubercle* 1981;62:13-30.
113 Velu S. A controlled comparison of streptomycin plus pyrazinamide and streptomycin plus pas in the retreatment of patients excreting isoniazid-resistant organisms. *Tubercle* 1964;45:144-59.
114 Wissenschaftliche Arbeitsgemeinschaft fur die Therapie von Lungenkrankheiten. Cooperative controlled trial of thiocarlide (DATC), PAS and bedrest alone in short-term single-drug treatment in retreated cavitary pulmonary tuberculosis. *Beitr Klin Erforsch Tuberk Lungenkr* 1969;139:115-39.
115 WHO Collaborating Centre for Tuberculosis Chemotherapy P. A study of two twice-weekly and a once-weekly continuation regimen of tuberculosis chemotherapy, including a comparison of two durations of treatment. II. Second report: the results at 36 months. *Tubercle* 1977;58:129-36.
116 WHO Collaborating Centre for Tuberculosis Chemotherapy P. A study of two twice-weekly and once-weekly continuation regimen of tuberculosis chemotherapy, including a comparison of two durations of treatment. 1. First report: the results at 18 months. *Tubercle* 1976;57:235-49.
117 Young PH, Sung CC, Sung CK. Comparison of retreatment regimens for pulmonary tuberculosis under programme condition. *Tuberc Respir Dis (Seoul)* 1981;28:95-109.

118 Zierski M, Bek E, Long MW, et al. Short-Course (6-Month) Cooperative Tuberculosis Study in Poland - Results 18 Months After Completion of Treatment. *Am Rev Respir Dis* 1980;122:879-89.
119 Zierski M, Bek E, Long MW, et al. Short-course (6-month) cooperative tuberculosis study in Poland: results 30 months after completion of treatment. *Am Rev Respir Dis* 1981;124:249-51.
