# Supplementary material for: What are the most efficacious treatment regimens for isoniazid-resistant tuberculosis? A systematic review and network meta-analysis
Source: Thorax. 2016 Jun 13;71(10):940–9. doi: 10.1136/thoraxjnl-2015-208262 (PMC5036252; doi:10.1136/thoraxjnl-2015-208262)
Supplement: Supplementary file 8 [file thoraxjnl-2015-208262supp_file8.pdf]

SUPPLEMENTARY FILE 8: Model fitting

Of the 59 studies of any INH resistance pattern 35 tested the same regimen category in every arm (Figure 1) and were thus excluded, as were an additional two studies with no events, leaving 22. Twelve studies had some of their treatment arms condensed together as they were of the same regimen category.

In an initial fixed-effects model the data network was reasonably well connected at its centre, but with some outlying regimen categories (Figure 3a). Some inconsistency was detected that produced high levels of variability in the estimates. Regimen category RIF ED<3 D<6m versus RIF ED<3 D=6m comparisons had particularly divergent observed versus predicted estimates with high deviances. Of the seven comparisons three studies had no events for regimen RIF ED<3 D=6m,[1-3] and one had an outlying odds ratio where regimen RIF ED<3 D=6m was worse than RIF ED<3 D<6m based on the raw data; this was the same outlying study identified in the pairwise analysis.[4] The results of this study were thought to be less clinically plausible than those of the others on which the summary estimate was based. Indeed, this study was the only RIF ED<3 D<6m versus RIF ED<3 D=6m comparison which included previously treated patients regardless of the length of such original treatment (which may explain relatively high level of negative outcomes) and, importantly, the single study where we deemed the dose of RIF low (below 450 mg or 10 mg/kg per day) in the RIF ED<3 D=6m arm, but not in the RIF ED<3 D<6m arm.[4]

On this basis, to improve model fit, this study was excluded from the network (Figure 3b). The main impact of this exclusion on the pairwise analysis was to reverse the direction of the overall effect estimate for this regimen pair (OR 0.36 (95% CI 0.17-0.76); Supplementary file 9).

Both fixed- and random-effects models were run on the new data network. The use of a random-effects model provided a marginally better fit than the use of a fixed-effects model (deviance information criteria score 230.6 versus 234.1). Given that the relative parsimony of the fixed-effects model avoided a reduction in power by not introducing perhaps unnecessary complexity its more certain estimates were not discarded. Results from both models are therefore presented in the main text.

References

1 Algerian Working Group/British Medical Research Council Cooperative Study. Short-course chemotherapy for pulmonary tuberculosis under routine programme conditions: a comparison of regimens of 28 and 36 weeks duration in Algeria. *Tubercle* 1991;72:88-100.

2 Singapore Tuberculosis Service British Medical Research Council. Long-Term Follow-Up of A Clinical Trial of Six-Month and Four-Month Regimens of Chemotherapy in the Treatment of Pulmonary Tuberculosis. *Am Rev Respir Dis* 1986;133:779-83.
3 Tam CM, Chan SL, Kam KM, et al. Rifapentine and isoniazid in the continuation phase of a 6-month regimen. Final report at 5 years: Prognostic value of various measures. *Int J Tuberc Lung Dis* 2002;6:3-10.
4 Mathew R, Rehman F, Santha T, et al. A controlled clinical trial of oral short-course regimens in the treatment of sputum-positive pulmonary tuberculosis. *Int J Tuberc Lung Dis* 1997;1:509-17.
