# Supplementary material for: What are the most efficacious treatment regimens for isoniazid-resistant tuberculosis? A systematic review and network meta-analysis
Source: Thorax. 2016 Jun 13;71(10):940–9. doi: 10.1136/thoraxjnl-2015-208262 (PMC5036252; doi:10.1136/thoraxjnl-2015-208262)

SUPPLEMENTARY FILE 9: Pairwise direct effects forest plots across all isoniazid resistance profiles when inconsistent study excluded

Pairwise direct effects forest plots for the three regimen pairs where such comparisons were possible and where the inconsistent study made that comparison. Regimen RIF ED<3 D<6m the baseline for plots a-b) and regimen RIF ED<3 D=6m for plot c). Regimen a) RIF ED<3 D=6m, b) RIF ED<3 Pr6 D=6m, c) RIF ED<3 Pr6 D=6m the comparator. In analysis c) study STS/BMRC had no events in either arm. Vertical solid line- null hypothesis. Vertical dotted line summary estimate. AWG/BMRC- Algerian Working Group/British Medical Research Council Cooperative Study, CI- confidence interval, EABMRC- East African British Medical Research Council Study, ECA/BMRC- East and Central African/British Medical Research Council, HKCS/BMRC- Hong Kong Chest Service/British Medical Research Council, OR- odds ratio, STS/BMRC- Singapore Tuberculosis Service/British Medical Research Council

a) RIF ED<3 D<6m versus RIF ED<3 D=6m

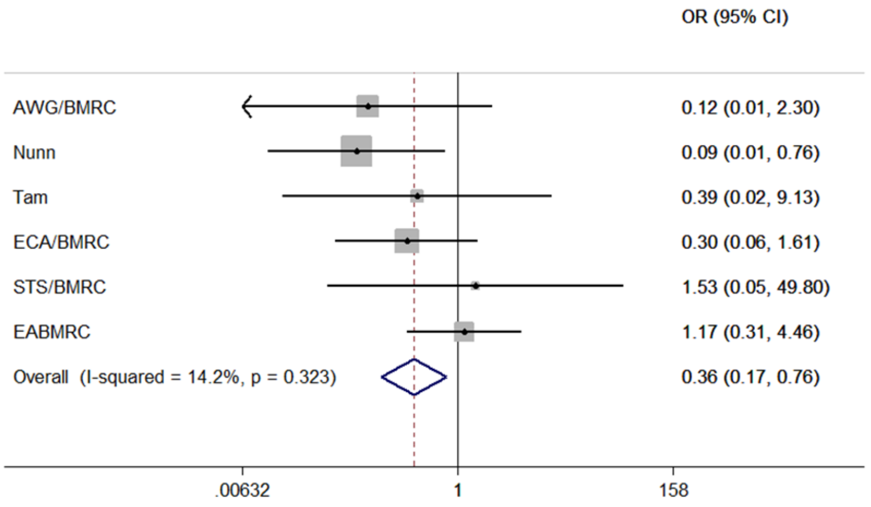

b) RIF ED<3 D<6m versus RIF ED<3 Pr6 D=6m

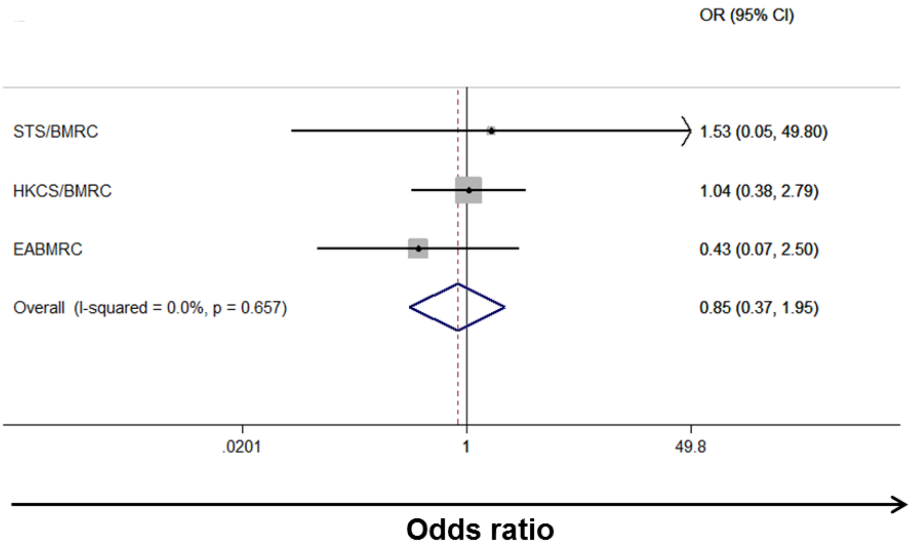

c) RIF ED<3 D=6m versus RIF ED<3 Pr6 D=6m

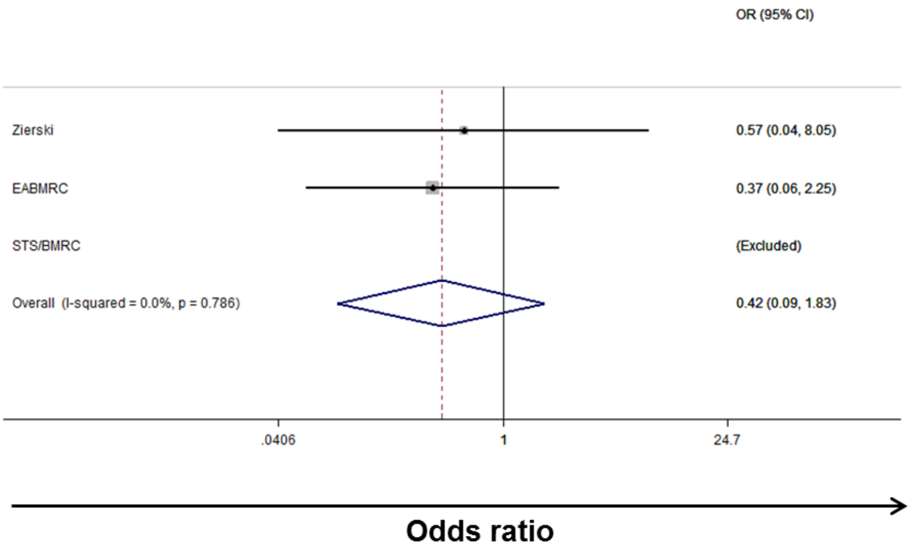

Supplement: Supplementary file 9 [file thoraxjnl-2015-208262supp_file9.pdf]
