# Supplementary material for: What are the most efficacious treatment regimens for isoniazid-resistant tuberculosis? A systematic review and network meta-analysis
Source: Thorax. 2016 Jun 13;71(10):940–9. doi: 10.1136/thoraxjnl-2015-208262 (PMC5036252; doi:10.1136/thoraxjnl-2015-208262)
Supplement: Supplementary file 10 [file thoraxjnl-2015-208262supp_file10.pdf]

## SUPPLEMENTARY FILE 10: SENSITIVITY ANALYSIS REGROUPING ONE ARM OF GIRLING *ET AL.*

### Odds ratios and relative ranks from fixed- and random-effects network meta-analyses across all isoniazid resistance profiles- one arm of Girling *et al.* 1991 recoded to regimen RIF ED<3 D=6m

Sensitivity analysis regrouping one treatment arm of Girling *et al.* 1991 [1] from regimen RIF ED≥3 Pr6 D=6m to RIF ED<3 D=6m to reflect uncertainty during categorisation. CrI- credible interval, OR- odds ratio

| | OR (95% CrI) | | Rank (95% CrI) | |
|---|---|---|---|---|
| Treatment | Fixed-effects | Random-effects | Fixed-effects | Random-effects |
| ED<3 D<6m | 3.66 (1.12-12.35) | 4.74 (0.53-59.53) | 11 (8-11) | 11 (4-11) |
| ED<3 D≥6m | 2.69 (1.28-5.85) | 2.69 (0.68-10.85) | 10 (8-11) | 9 (5-11) |
| ED≥3 D≥6m | 0.95 (0.37-2.41) | 1.01 (0.16-6.73) | 6 (3-9) | 6 (2-10) |
| RIF ED<3 D<6m | 1.07 (0.57-2.05) | 1.45 (0.50-5.95) | 6 (4-9) | 7 (4-10) |
| RIF ED<3 D=6m | 0.56 (0.26-1.20) | 0.62 (0.18-2.19) | 3 (2-6) | 4 (1-8) |
| RIF ED<3 D>6m | 0.20 (0.03-1.21) | 0.22 (0.01-3.87) | 1 (1-7) | 1 (1-10) |
| RIF ED<3 Pr6 D=6m | baseline | baseline | 6 (3-9) | 6 (2-9) |
| RIF ED<3 Pr6 D>6m | 1.04 (0.40-2.67) | 0.88 (0.12-5.53) | 6 (3-10) | 5 (1-10) |
| RIF ED≥3 D<6m | 1.68 (0.59-4.76) | 1.99 (0.38-12.28) | 9 (4-10) | 8 (3-11) |
| RIF ED≥3 Pr6 D=6m | 0.83 (0.02-11.10) | 0.97 (0.01-35.60) | 5 (1-11) | 5 (1-11) |
| RIF ED≥3 Pr6 D>6m | 0.33 (0.12-0.84) | 0.48 (0.08-4.67) | 2 (1-4) | 3 (1-9) |

**Odds ratios and relative ranks from fixed- and random-effects network meta-analyses across all isoniazid resistance profiles- one arm of Girling *et al.* 1991 recoded to regimen RIF ED<3 Pr6 D=6m**

Sensitivity analysis regrouping one treatment arm of Girling *et al.* 1991 [1] from regimen RIF ED≥3 Pr6 D=6m to RIF ED<3 Pr6 D=6m (and thereby losing the study from the network, due to both treatment arms being grouped the same) to reflect uncertainty during categorisation. CrI- credible interval, OR- odds ratio

| | OR (95% CrI) | | Rank (95% CrI) | |
|---|---|---|---|---|
| Treatment | Fixed-effects | Random-effects | Fixed-effects | Random-effects |
| ED<3 D<6m | 3.44 (1.04-11.86) | 4.47 (0.42-67.25) | 11 (8-11) | 11 (4-11) |
| ED<3 D≥6m | 2.53 (1.18-5.59) | 2.50 (0.56-11.64) | 10 (8-11) | 9 (5-11) |
| ED≥3 D≥6m | 0.91 (0.35-2.33) | 0.95 (0.13-7.25) | 6 (3-9) | 6 (2-10) |
| RIF ED<3 D<6m | 0.99 (0.51-1.96) | 1.35 (0.42-6.57) | 6 (4-9) | 7 (4-10) |
| RIF ED<3 D=6m | 0.48 (0.20-1.14) | 0.53 (0.12-2.39) | 3 (2-6) | 3 (1-8) |
| RIF ED<3 D>6m | 0.17 (0.02-1.08) | 0.19 (0.01-4.19) | 1 (1-6) | 1 (1-10) |
| RIF ED<3 Pr6 D=6m | baseline | baseline | 6 (3-9) | 6 (2-10) |
| RIF ED<3 Pr6 D>6m | 1.01 (0.38-2.62) | 0.84 (0.11-6.04) | 6 (3-10) | 5 (1-11) |
| RIF ED≥3 D<6m | 1.58 (0.54-4.53) | 1.88 (0.32-13.36) | 9 (4-10) | 8 (3-11) |
| RIF ED≥3 Pr6 D=6m | 0.77 (0.02-10.43) | 0.91 (0.01-41.50) | 5 (1-11) | 5 (1-11) |
| RIF ED≥3 Pr6 D>6m | 0.31 (0.12-0.81) | 0.46 (0.06-5.26) | 2 (1-4) | 3 (1-10) |

## References

1 Girling DJ, Chan SL. Controlled trial of 2, 4, and 6 months of pyrazinamide in 6-month, three-times-weekly regimens for smear-positive pulmonary tuberculosis, including an assessment of a combined preparation of isoniazid, rifampin, and pyrazinamide: Results at 30 months. *Am Rev Respir Dis* 1991;143:700-6.
