# Supplementary material for: What are the most efficacious treatment regimens for isoniazid-resistant tuberculosis? A systematic review and network meta-analysis
Source: Thorax. 2016 Jun 13;71(10):940–9. doi: 10.1136/thoraxjnl-2015-208262 (PMC5036252; doi:10.1136/thoraxjnl-2015-208262)
Supplement: Supplementary file 11 [file thoraxjnl-2015-208262supp_file11.pdf]

SUPPLEMENTARY FILE 11: SENSITIVITY ANALYSIS- RIFAMPICIN DOSE

Treatment regimen categorisation

Regimen categorisation flow chart for sensitivity analysis, with additional classification by dose of RIF (10mg/kg threshold). Categories present in network presented in bold. *Levamisole and diphenyl thiourea compound SU 1906 not counted as effective protection. INH- isoniazid, RIF- rifampicin.

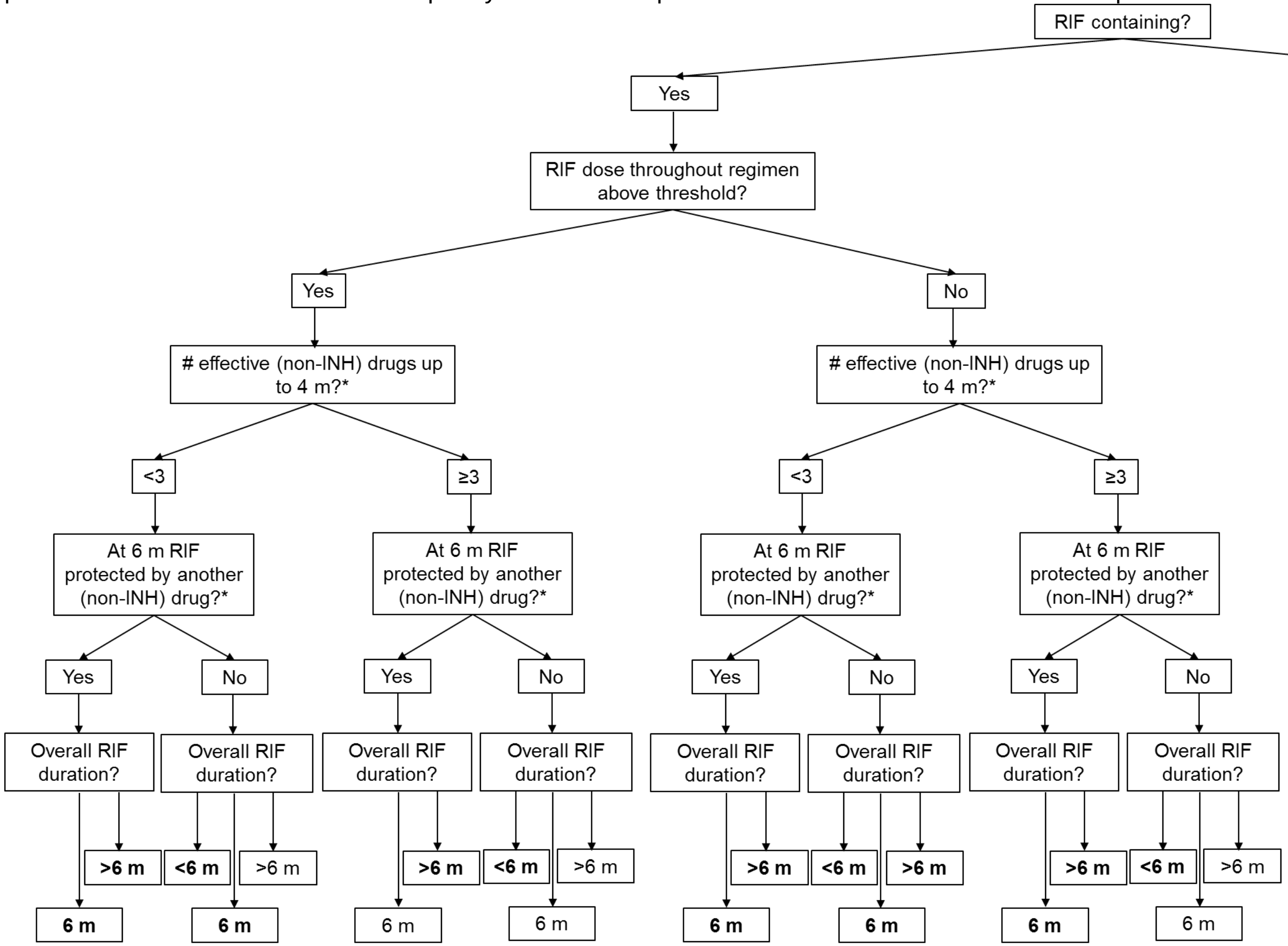

Regimen category codes

Encoded regimen categories present in the network where rifampicin dose was taken into account (10mg/kg threshold). 4m- 4 months, 6m- 6 months, D</=/>6m- overall RIF duration or duration of the entire regimen if RIF not present, ED- effective drugs, N/A- not applicable, Pr6- protected at 6 months, RIF- rifampicin, RIF<10- RIF dose below threshold

**RIF containing?**	**RIF dose above threshold throughout study (<10)?**	**# of effective drugs (ED) up to 4m?**	**At 6m RIF protected (Pr6) by another drug?**	**Overall (RIF) duration (D)**	**Code**
No	N/A	<3	N/A	<6m	ED<3 D<6m
No	N/A	<3	N/A	≥6m	ED<3 D≥6m
No	N/A	≥3	N/A	≥6m	ED≥3 D≥6m
Yes	No	<3	No	<6m	RIF<10 ED<3 D<6m
Yes	No	<3	No	6m	RIF<10 ED<3 D=6m
Yes	No	<3	No	>6m	RIF<10 ED<3 D>6m
Yes	No	<3	Yes	6m	RIF<10 ED<3 Pr6 D=6m
Yes	No	<3	Yes	>6m	RIF<10 ED<3 Pr6 D>6m
Yes	No	≥3	No	<6m	RIF<10 ED≥3 D<6m
Yes	No	≥3	Yes	6m	RIF<10 ED≥3 Pr6 D=6m
Yes	No	≥3	Yes	>6m	RIF<10 ED≥3 Pr6 D>6m
Yes	Yes	<3	No	<6m	RIF ED<3 D<6m
Yes	Yes	<3	No	6m	RIF ED<3 D=6m
Yes	Yes	<3	Yes	6m	RIF ED<3 Pr6 D=6m
Yes	Yes	<3	Yes	>6m	RIF ED<3 Pr6 D>6m
Yes	Yes	≥3	No	<6m	RIF ED≥3 D<6m
Yes	Yes	≥3	Yes	>6m	RIF ED≥3 Pr6 D>6m

Data network

Data network utilising the regimen categorisation presented above. Thickness of lines and numbers indicate the number of studies making this comparison. *One study arm classifiable as RIF<10 ED≥3 Pr6 D=6m, RIF<10 ED<3 Pr6 D=6m or RIF<10 ED<3 D=6m compared to RIF<10 ED<3 D=6m; listed as RIF<10 ED≥3 Pr6 D=6m as per this analysis.

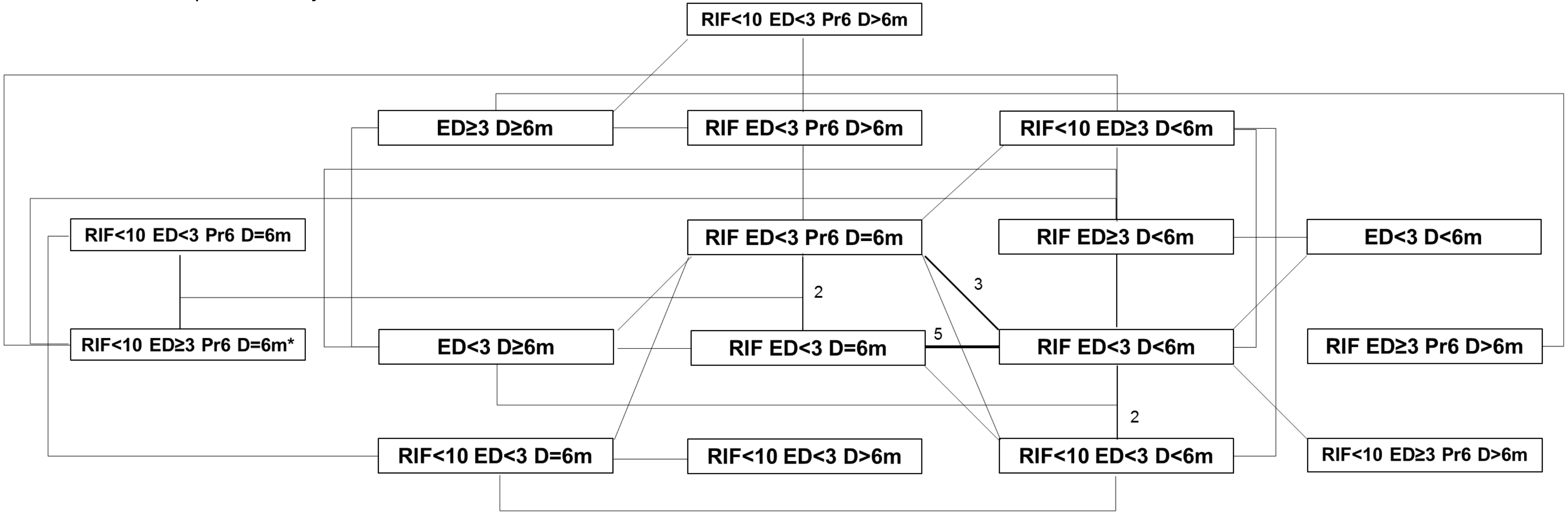

Odds ratios and relative ranks from fixed- and random-effects network meta-analyses across all isoniazid resistance profiles

Sensitivity analysis further dividing the regimen groups into additional categorisations on the basis of having a low versus an acceptable rifampicin dose. CrI- credible interval, OR- odds ratio

	OR (95% CrI)		Rank (95% CrI)	
Treatment	Fixed-effects	Random-effects	Fixed-effects	Random-effects
ED<3 D<6m	4.72 (1.33-18.48)	5.39 (0.53-69.49)	17 (13-17)	17 (7-17)
ED<3 D≥6m	2.67 (1.23-6.06)	2.48 (0.59-10.27)	15 (12-17)	14 (7-17)
ED≥3 D≥6m	0.68 (0.19-2.20)	0.67 (0.08-5.55)	7 (2-14)	7 (2-15)
RIF<10 ED<3 D<6m	1.54 (0.54-4.34)	1.89 (0.39-12.97)	13 (6-16)	13 (5-17)
RIF<10 ED<3 D=6m	0.44 (0.01-5.47)	0.44 (0.01-8.83)	5 (1-17)	5 (1-17)
RIF<10 ED<3 D>6m	0.15 (0.00-3.18)	0.15 (0.00-7.77)	2 (1-15)	2 (1-16)
RIF<10 ED<3 Pr6 D=6m	0.42 (0.03-3.84)	0.41 (0.02-6.80)	5 (1-16)	5 (1-16)
RIF<10 ED<3 Pr6 D>6m	1.01 (0.29-3.31)	0.97 (0.07-12.33)	10 (4-16)	9 (2-17)
RIF<10 ED≥3 D<6m	0.94 (0.20-3.66)	1.14 (0.15-9.79)	9 (2-16)	10 (2-17)
RIF<10 ED≥3 Pr6 D=6m	0.56 (0.04-4.88)	0.61 (0.03-10.32)	6 (1-16)	7 (1-17)
RIF<10 ED≥3 Pr6 D>6m	0.81 (0.18-3.68)	0.96 (0.07-16.37)	8 (2-16)	9 (1-17)
RIF ED<3 D<6m	1.13 (0.56-2.36)	1.34 (0.42-5.89)	11 (6-14)	11 (6-15)
RIF ED<3 D=6m	0.55 (0.22-1.37)	0.54 (0.12-2.42)	6 (2-11)	6 (2-12)
RIF ED<3 Pr6 D=6m	baseline	baseline	10 (5-14)	9 (4-15)
RIF ED<3 Pr6 D>6m	0.58 (0.18-1.71)	0.54 (0.07-3.76)	6 (2-13)	6 (1-15)
RIF ED≥3 D<6m	2.52 (0.74-9.14)	2.59 (0.38-19.59)	15 (9-17)	14 (6-17)
RIF ED≥3 Pr6 D>6m	0.22 (0.06-0.75)	0.22 (0.01-4.05)	3 (1-7)	3 (1-14)
