# Supplementary material for: What are the most efficacious treatment regimens for isoniazid-resistant tuberculosis? A systematic review and network meta-analysis
Source: Thorax. 2016 Jun 13;71(10):940–9. doi: 10.1136/thoraxjnl-2015-208262 (PMC5036252; doi:10.1136/thoraxjnl-2015-208262)
Supplement: Supplementary file 12 [file thoraxjnl-2015-208262supp_file12.pdf]

SUPPLEMENTARY FILE 12: ISONIAZID MONORESISTANCE ONLY

Data network

Data network restricted to patients with isoniazid monoresistant disease only. Thickness of lines and numbers indicate the number of studies making this comparison. *One study arm classifiable as RIF ED≥3 Pr6 D=6m, RIF ED<3 Pr6 D=6m or RIF ED<3 D=6m compared to RIF ED<3 D=6m; here listed as RIF ED≥3 Pr6 D=6m as per this analysis.

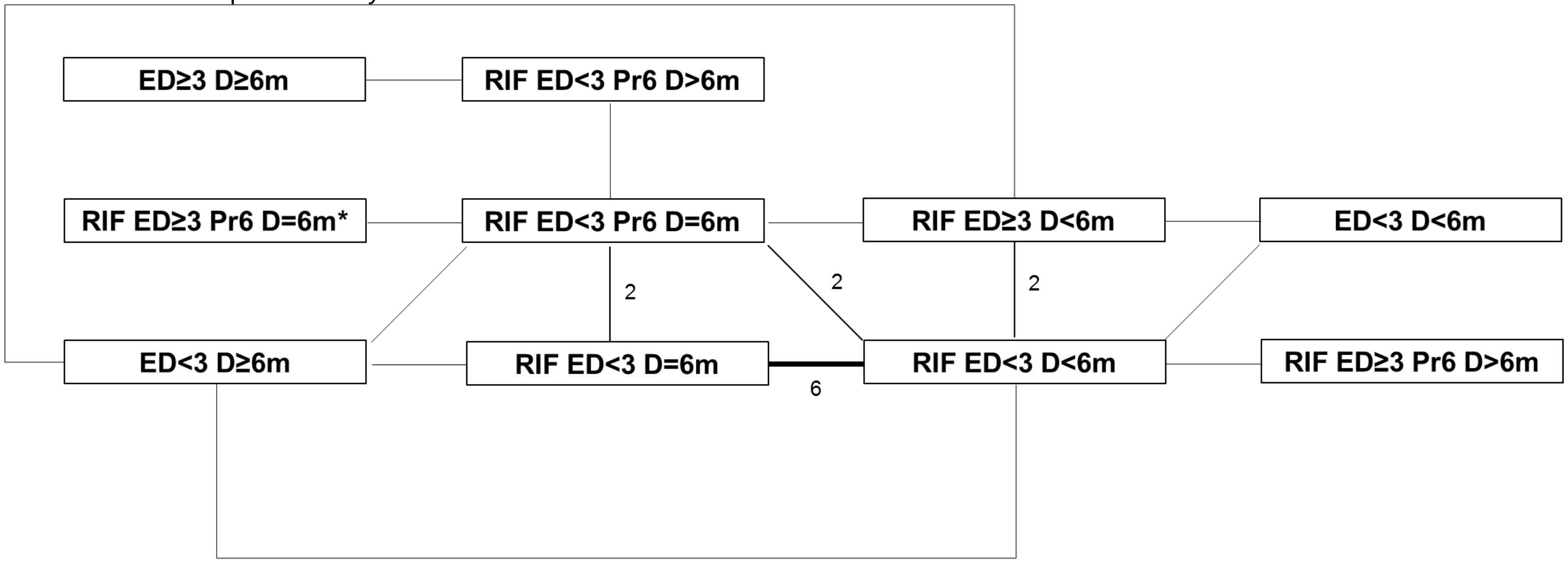

Odds ratios and relative ranks from fixed- and random-effects network meta-analyses

Sensitivity analysis restricting to studies where isoniazid monoresistance was described. CrI- credible interval, OR- odds ratio

	OR (95% CrI)		Rank (95% CrI)	
Treatment	Fixed-effects	Random-effects	Fixed-effects	Random-effects
ED<3 D<6m	15.64 (2.18-122.30)	18.14 (0.62-730.00)	10 (7-10)	10 (4-10)
ED<3 D≥6m	3.49 (1.14-12.65)	3.12 (0.28-23.50)	8 (4-10)	7 (2-10)
ED≥3 D≥6m	0.70 (0.10-4.71)	0.70 (0.01-56.69)	2 (1-8)	3 (1-10)
RIF ED<3 D<6m	2.80 (0.90-9.88)	3.40 (0.58-30.14)	7 (4-9)	7 (4-9)
RIF ED<3 D=6m	0.77 (0.20-2.93)	0.83 (0.10-6.31)	3 (1-6)	3 (1-7)
RIF ED<3 Pr6 D=6m	baseline	baseline	4 (1-6)	4 (1-7)
RIF ED<3 Pr6 D>6m	1.03 (0.18-6.01)	1.02 (0.04-27.79)	4 (1-9)	4 (1-10)
RIF ED≥3 D<6m	6.57 (1.25-38.16)	6.87 (0.41-115.20)	9 (5-10)	9 (3-10)
RIF ED≥3 Pr6 D=6m	0.62 (0.04-21.17)	0.63 (0.01-52.19)	2 (1-10)	3 (1-10)
RIF ED≥3 Pr6 D>6m	2.02 (0.32-13.62)	2.45 (0.07-118.00)	6 (1-9)	6 (1-10)
