# Supplementary material for: What are the most efficacious treatment regimens for isoniazid-resistant tuberculosis? A systematic review and network meta-analysis
Source: Thorax. 2016 Jun 13;71(10):940–9. doi: 10.1136/thoraxjnl-2015-208262 (PMC5036252; doi:10.1136/thoraxjnl-2015-208262)

SUPPLEMENTARY FILE 13: Funnel plots

Pairwise direct effects forest plots for the four regimen pairs where such comparisons were possible. Regimen RIF ED<3 D<6m the baseline for plots a-c) and regimen RIF ED<3 D=6m for plot d). Regimen RIF ED<3 D=6m (a), RIF ED<3 Pr6 D=6m (b, d) and RIF ED≥3 D<6m (c) the comparator. In analysis d) study STS/BMRC had no events in either arm. se(logOR)- standard error of the log(odds ratio)

a) RIF ED<3 D<6m versus RIF ED<3 D=6m

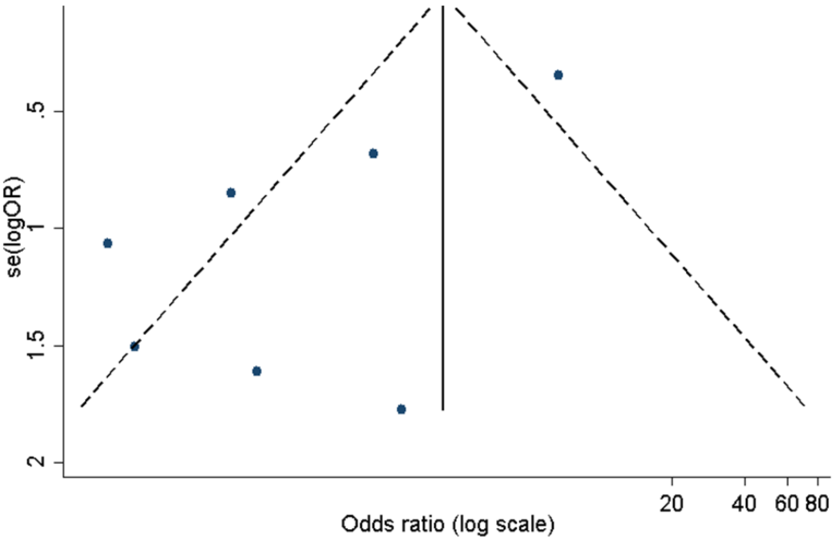

b) RIF ED<3 D<6m versus RIF ED<3 Pr6 D=6m

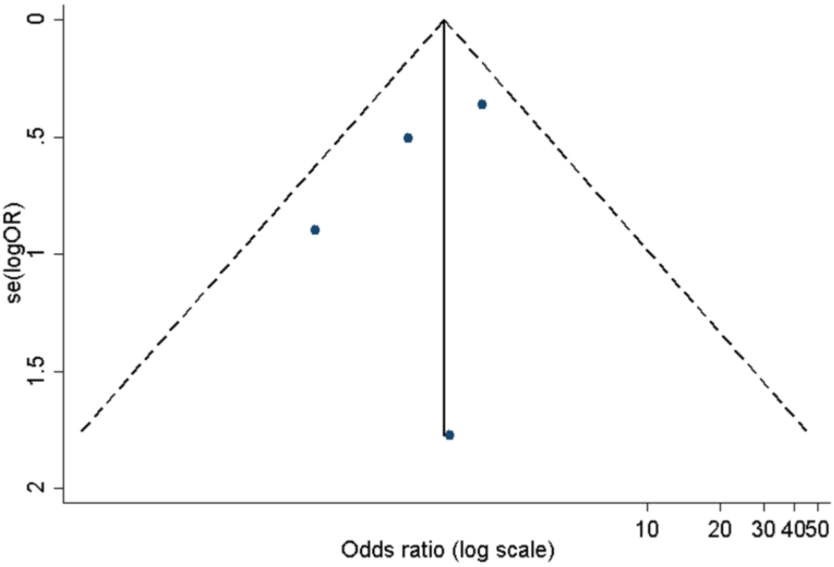

c) RIF ED<3 D<6m versus RIF ED≥3 D<6m

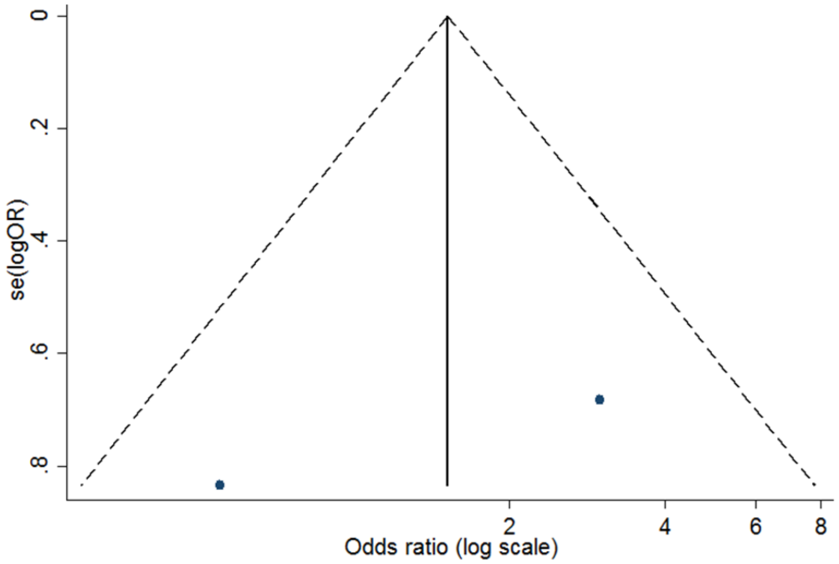

d) RIF ED<3 D=6m versus RIF ED<3 Pr6 D=6m

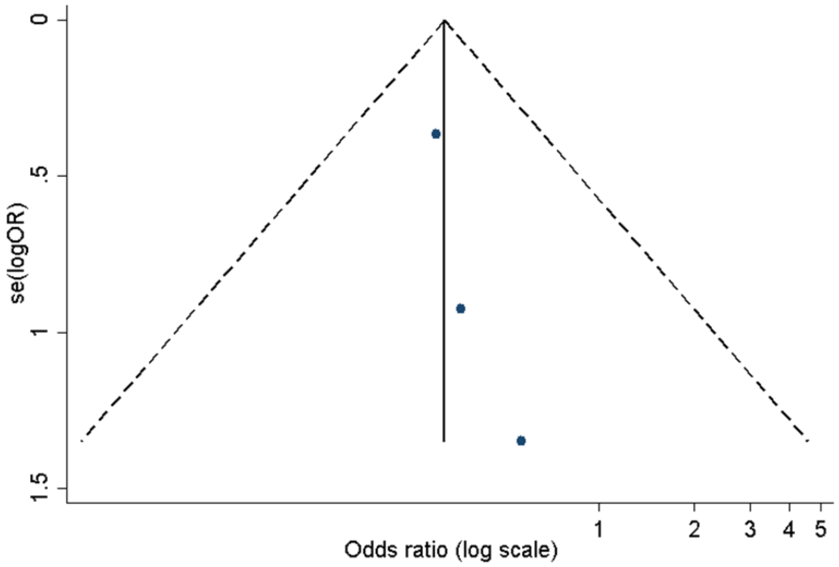

Supplement: Supplementary file 13 [file thoraxjnl-2015-208262supp_file13.pdf]
