# Supplementary material for: What are the most efficacious treatment regimens for isoniazid-resistant tuberculosis? A systematic review and network meta-analysis
Source: Thorax. 2016 Jun 13;71(10):940–9. doi: 10.1136/thoraxjnl-2015-208262 (PMC5036252; doi:10.1136/thoraxjnl-2015-208262)
Supplement: Supplementary file 14 [file thoraxjnl-2015-208262supp_file14.pdf]

SUPPLEMENTARY FILE 14: Comparison to Menzies reviews

Menzies *et al.* published two systematic reviews in 2009, one of which sought to determine the effectiveness of the WHO recommended retreatment regimen at that time (2 months of INH EMB RIF STM PZA followed by one of INH EMB RIF PZA and then five of INH EMB RIF) in previously treated patients or those with INH monoresistance.[1] The second looked at the impact of the duration and intermittency of RIF, stratifying by different resistance patterns, including INH monoresistance and INH-STM resistance.[2]

Although we considered all the studies from both Menzies publications for inclusion in our review, the final list of publications differed substantially between our work and theirs, largely due to three criteria- 1) the temporal (1965-June 2008) and language (English, French, Spanish) inclusion criteria put in place by Menzies, 2) their exclusion of studies that utilised rifapentine or rifabutin, or regimens dosed once weekly or of a single drug alone, 3) our exclusion of studies where the INH monoresistant population was not considered sufficiently 'pure'. We agree with Menzies that regimens dosed once weekly or of a single drug alone are likely to be inadequate, but they added statistical strength to our data network, even if they would not be used in clinical practice. Our more up-to-date search also identified two extra studies [3, 4] and one study update [5] since June 2008. Indeed, in our review we included 32 extra studies versus the first Menzies paper,[1] and 38 versus the second when studies of INH monoresistance or non-MDR poly drug resistance were described.[2] Five studies included by the first Menzies paper were not considered eligible for our review (one did not break down treatment outcomes by regimen, one included a number of patients with MDR disease imbalanced between treatment arms whose outcomes could not be separated from those with INH monoresistant disease, and three were not randomised by treatment regimen or randomisation was broken during the trial)[6-10] and nine included by the second (five did not report treatment outcomes for patients with INH monoresistant strains by treatment regimen, three were either not randomised by treatment regimen or randomisation was broken during the trial, one did not report baseline resistance patterns),[8, 10-18] with two in common.

Unlike with meta-regression, our analysis technique additionally involved the loss of a number of studies from the final network due to all treatment arms having the same regimen under our grouping system (and a few were additionally lost due to having no events in any arm). However, the Menzies studies used models treating each study arm as an independent cohort, breaking within-study comparisons. Therefore if certain treatments were given to higher/lower incidence populations the resulting estimates of treatment differences could be biased (a limitation admitted by the authors). There is therefore a trade-off between

the inclusion of studies numbers and methodological rigour.

References

1 Menzies D, Benedetti A, Paydar A, et al. Standardized treatment of active tuberculosis in patients with previous treatment and/or with mono-resistance to isoniazid: a systematic review and meta-analysis. *PLoS Med* 2009;6:e1000150.
2 Menzies D, Benedetti A, Paydar A, et al. Effect of duration and intermittency of rifampin on tuberculosis treatment outcomes: a systematic review and meta-analysis. *PLoS Med* 2009;6:e1000146.
3 Park SH, Yang SK, Yang DH, et al. Prospective Randomized Trial of Six-Month versus Nine-Month Therapy for Intestinal Tuberculosis. *Antimicrob Agents Chemother* 2009;53:4167-71.
4 Swaminathan S, Narendran G, Venkatesan P, et al. Efficacy of a 6-month versus 9-month intermittent treatment regimen in HIV-infected patients with tuberculosis: A randomized clinical trial. *Am J Respir Crit Care Med* 2010;181:01.
5 Nunn AJ, Jindani A, Enarson DA. Results at 30 months of a randomised trial of two 8-month regimens for the treatment of tuberculosis. *Int J Tuberc Lung Dis* 2011;15:741-5.
6 Castelo A, Jardim JRB, Goihman S, et al. Comparison of Daily and Twice-Weekly Regimens to Treat Pulmonary Tuberculosis. *Lancet* 1989;2:1173-6.
7 Aziz A, Ishaq M, Jaffer NA, et al. Clinical-Trial of 2 Short-Course (6-Month) Regimens and A Standard Regimen (12-Month) Chemotherapy in Retreatment of Pulmonary Tuberculosis in Pakistan - Results 18 Months After Completion of Treatment (Lahore Tuberculosis Study). *Am Rev Respir Dis* 1986;134:1056-61.
8 De Figueiredo FP, Alves BA, Laborne Valle JH, et al. Short Duration Chemo Therapy of Pulmonary Tuberculosis A Pilot Trial. *Bull Int Union Tuberc* 1974;49:382-8.
9 Hong Kong Tuberculosis Treatment Service, Brompton Hospital, British Medical Ressearch Council Investigation. A Controlled Clinical Trial of Daily and Intermittent Regimens of Rifampicin Plus Ethambutol in the Re Treatment of Patients with Pulmonary Tuberculosis in Hong-Kong. *Tubercle* 1974;55:1-27.
10 Girling DJ. A controlled clinical comparison of 6 and 8 months of antituberculosis chemotherapy in the treatment of patients with silicotuberculosis in Hong Kong. *Am Rev Respir Dis* 1991;143:262-7.
11 ANGEL JH, Campbell IA, Dyson A, et al. Short Course Chemo Therapy in Pulmonary Tuberculosis A Controlled Trial by the British Thoracic and Tuberculosis Association. *Lancet* 1976;2:1102-4.
12 British Thoracic Society and Tuberculosis Association. Short-course chemotherapy in pulmonary tuberculosis. A controlled trial by the British Thoracic and Tuberculosis Association. *Lancet* 1975;1:119-24.
13 Felten MK. Importance of rifampicin in combined daily/intermittent chemotherapy for tuberculosis. *S Afr Med J* 1989;75:524-6.
14 Gonzalez-Montaner LJ, Natal S, Yongchaiyud P, et al. Rifabutin for the treatment of newly-diagnosed pulmonary tuberculosis: a multinational, randomized, comparative study versus Rifampicin. Rifabutin Study Group. *Tuber Lung Dis* 1994;75:341-7.
15 Macnab MF, Bohmer PD, Seager JR. Evaluation of the 3-drug combination, Rifater, versus 4-drug therapy in the ambulatory treatment of tuberculosis in Cape Town. *S Afr Med J* 1994;84:325-8.
16 Mohanty KC, Dhamgaye TM. Controlled trial of ciprofloxacin in short-term chemotherapy for pulmonary tuberculosis. *Chest*104:1993.
17 Pedral-Sampaio DB, Netto EM, Brites C, et al. Use of Rhu-GM-CSF in pulmonary tuberculosis patients: results of a randomized clinical trial. *Braz J Infect Dis* 2003;7:245-52.

18 Perriens JH, Stlouis ME, Mukadi YB, et al. Pulmonary Tuberculosis in Hiv-Infected Patients in Zaire - A Controlled Trial of Treatment for Either 6 Or 12 Months. *N Engl J Med* 1995;332:779-84.
